# Linear and Nonlinear Optical Properties of Molecules from Real-Time Propagation Based on the Bethe–Salpeter Equation

**DOI:** 10.1021/acs.jctc.5c01246

**Published:** 2025-09-25

**Authors:** Štěpán Marek, Jan Wilhelm

**Affiliations:** Regensburg Center for Ultrafast Nanoscopy and Institute of Theoretical Physics, 9147 University of Regensburg, Regensburg D-93040, Germany

## Abstract

We present a real-time propagation method for computing linear and nonlinear optical properties of molecules based on the Bethe–Salpeter equation. The method follows the time evolution of the one-particle density matrix under an external electric field. We include electron–electron interaction effects through a self-energy model based on the screened exchange approximation. Quasiparticle energies are taken from a prior *GW* calculation to construct the effective single-particle Hamiltonian, and we represent all operators and wave functions in an atom-centered Gaussian basis. We benchmark the accuracy of the real-time propagation against the standard linear-response Bethe–Salpeter equation by using a set of organic molecules. We find very good agreement when computing linear-response isotropic polarizability spectra from both approaches with a mean absolute deviation of 30 meV in peak positions. Beyond linear response, we simulate second harmonic generation and optical rectification in a noncentrosymmetric molecule. We foresee broad applicability of real-time propagation based on the Bethe–Salpeter equation for the study of linear and nonlinear optical properties of molecules, as the method has a computational cost similar to that of time-dependent density functional theory with hybrid functionals.

## Graphical abstract

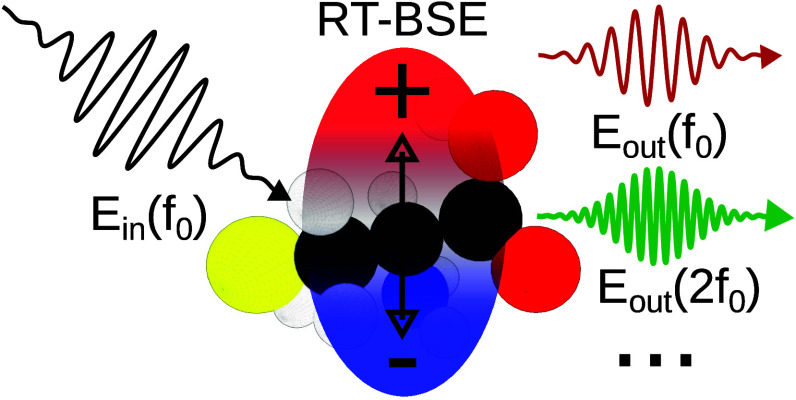

## 1 Introduction

Recent advances in the optical microscopy,^1,2^ scanning tunnelling microscopy^3^ and laser pulse control^4,5^ have significantly increased the amount of experimentally accessible information about the light-driven excitations of matter. In many investigations, nonlinear optical response^6–9^ properties become important. This calls for theory of nonlinear optical responses which could help to gain further insights into the mechanism behind the observed nonlinear optical phenomena.

Commonly used methods for the theoretical description of optical excitations and absorption spectra from first principles include coupled clusters based methods,^10–15^ time-dependent density functional theory^16–23^ (TDDFT) and the *GW*-Bethe–Salpeter equation (*GW*-BSE) approach.^16,24–30^ The computational cost of canonical implementations of coupled clusters methods scales steeply with the size of the system *N*, from *N* ^5^ to *N* ^6^.^31^ For larger systems, it can therefore become very expensive to execute coupled clusters calculations. For TDDFT, the scaling is reduced, but when using local and semilocal approximate exchange-correlation potentials, the excitonic effects, stemming from the screened Coulomb interaction between the excited charge carriers, are described inaccurately or may be missing entirely.^17^ This limitation in TDDFT can be improved by using more advanced functionals, such as tuned hybrid functionals.^16^ However, developing a single functional that reliably describes excited-state phenomena across a broad range of matter, including metals, atomically thin semiconductors, and molecules, remains a major challenge. Furthermore, the computational cost of hybrid functionals is significantly higher than the cost of local or semilocal functionals.

Compared to TDDFT with local or semilocal functionals, the *GW*–BSE approach provides a more accurate description of excitonic effects and optical spectra, as *GW*-BSE naturally includes the screened Coulomb interaction between the excited charge carriers.^24,32^ Common implementations of *GW*-BSE^32–39^ employ the Casida framework to study electronic excitations in the linear response limit, so for relatively weak driving electric fields. Due to advances in laser spectroscopy,^40^ also nonlinear optical excitations are commonly studied, which go beyond this Casida framework. Specific nonlinear optical properties, in particular low-order responses in second and third order, can be computed in frequency domain in *GW*-BSE,^41–43^ but a general analytical expression to arbitrary order in the response remains elusive at present.

In contrast, the real-time approach for studying electronic excitations propagates the electronic many-body system in real time, and Fourier transforms the electric dipole to obtain nonlinear optical responses. In a Green’s function framework, the exact propagation can be described by the Kadanoff-Baym equations^44–49^ (KBEs). The self-energy in KBEs is time nonlocal, which increases the computational effort considerably.

In order to reduce the computational effort of KBEs, we apply the real-time Bethe–Salpeter equation approach^50–55^ (RT-BSE), which uses time local approximations for the self-energy to arrive at algorithms with computational cost comparable to RT-TDDFT with hybrid functionals (which is typically *N* ^4^ or, when using sparse matrix methods, *N* ^2^). Specifically, the screened exchange^56–58^ (SEX) approximation is applied.

RT-BSE allows for evaluation of nonlinear effects, such as second harmonic generation or optical rectification.^40,52^ In the time propagation scheme, the nonlinear effects are present to arbitrary order. For these effects, a description of excitons is again crucial for accurate determination of the absorption/emission spectra.

We report an implementation of the RT-BSE approach for molecular systems in the CP2K^59^ program. We performed a benchmark on standard test set of organic molecules^60^ comparing excitation energies from the real-time (RT) approach to the ones from the linear response^24,32,50,61^ (LR) approach. Note that LR-BSE and RT-BSE approaches are equivalent in the linear response limit (see ref 50 or Supporting Information 1 (SI 1)). Going beyond the linear response limit, we study nonlinear optical phenomena in molecules, specifically we investigate the emergence of second harmonic generation^40^ and optical rectification in the noncentrosymmetric cysteine molecule.

## 2 Equation of Motion for the Density Operator within the Real-Time Bethe–Salpeter Approach

To study the interaction of a many-electron system with light, we follow the RT-BSE approach described in ref 50, which employs the von Neumann equation

$$\frac{\partial \mathrm{\rho ̂}\left(t\right)}{\partial t}=\frac{-i}{\hbar }\left[{Ĥ}^{\mathrm{eff}}\left(t\right),\mathrm{\rho ̂}\left(t\right)\right]$$ 1

to compute the time evolution of the one-particle density operator ρ̂(*t*). The time-dependent effective one-particle Hamiltonian is

$${Ĥ}^{\mathrm{eff}}\left(t\right)={\mathrm{h̑}}^{G_{0}W_{0}}+Û\left(t\right)+{\mathrm{V̂}}^{H}\left[\mathrm{\rho ̂}\left(t\right)\right]-{\mathrm{V̂}}^{H}\left[\mathrm{\rho ̂}\left(0\right)\right]+{\mathrm{\Sigma ̂}}^{\mathrm{SEX}}\left[\mathrm{\rho ̂}\left(t\right)\right]-{\mathrm{\Sigma ̂}}^{\mathrm{SEX}}\left[\mathrm{\rho ̂}\left(0\right)\right]$$ 2

where *ĥ* ^*G*_0_ *W*_0_^ is the static effective one-particle Hamiltonian operator obtained from a preceding DFT plus *G*_0_ *W*_0_ calculation,^62,63^

h^G0W0=∑n|ψn⟩ϵnG0W0⟨ψn| 3

where |ψ_*n*_⟩ is a Kohn–Sham DFT (KS-DFT) orbital, and ϵ_*n*_^*G*_0_ *W*_0_^ is the corresponding *G*_0_ *W*_0_ quasiparticle energy. Note that the RT-BSE scheme can also be started from *GW* schemes including self-consistency, for example from *GW* with eigenvalue-self-consistency in *G* (ev*GW*_0_) which might align better with quasiparticle energies from higher-level theories.^63–66^

*V* ^^H^ is the Hartree mean-field operator and Σ̂^SEX^ is the SEX self-energy operator, which are both specified in more detail when rewriting the equation of motion in an atomic Gaussian basis. In contrast to ref 50., the Coulomb-hole self-energy is not included, since it is exactly canceled in eq 2 for static screened potential fixed at *G*_0_ *W*_0_ or ev*GW*_0_ screening. The dynamics starts at *t* = 0, and ρ̂(0) is the initial density operator,

$$\mathrm{\rho ̂}\left(0\right)=\underset{n}{\sum }\left|\psi_{n}\right\rangle f_{n}\left\langle \psi_{n}\right|$$ 4

where *f*_*n*_ ∈{0, 1} is the occupation of the Kohn–Sham orbital *n* from the KS-DFT calculation. Note that eq 1 does not contain damping terms,^67^ which are commonly introduced in studies of ultrafast electron dynamics in solids to mimic electron–electron and electron–phonon scattering.

*Û*(*t*) is the time-dependent external field operator. In order to excite the system, we implemented two options. First option is to apply an external time dependent potential *Û*(*t*) in form of electric field in length gauge^68,69^

Û(t)=eE(t)ϵ·r̂ 5

where *E*(*t*) is the time-dependent electric-field amplitude, **ϵ** is the electric field polarization direction and *r̂* is the position operator.

The second option is the delta kick,^50,68,70^ which is described in more detail in the SI 2. In essence, the delta kick transforms the equilibrium density matrix ρ̂(0) to a nonequilibrium one via unitary transformation

$$\mathrm{\rho ̂}\left(0\right)\to {\mathrm{\rho ̂}}^{\prime }=e^{\left(-ie/\hbar \right)I\epsilon \cdot \mathrm{r̂}}\mathrm{\rho ̂}\left(0\right)e^{\left(ie/\hbar \right)I\epsilon \cdot \mathrm{r̂}}$$ 6

where *I* is the scale of the kick and **ϵ** is the polarization of the kick. This transformation is associated with electric field with profile ***E*** (*t*) = *I* **ϵ**δ­(*t*), where δ­(*t*) is the Dirac delta function and hence the name of the excitation scheme.

We compute the observables from the Fourier transform of the time-dependent dipole moment associated with the dynamics of the density matrix^70,71^ (see SI 3 for details). The Fourier transform of the dipole moments is refined using the Padé approximant interpolation,^72–75^ which allows the use of a fine frequency grid.

## 3 Real-Time Bethe–Salpeter in a Gaussian Basis

We represent all operators appearing in the effective one-particle Hamiltonian (eq 2) as matrices in the basis of atom-centered Gaussian orbitals |ϕ_μ_⟩. Since this is a nonorthonormal basis set, the overlap matrix ***S*** with elements *S*_*μν*_ = ⟨ϕ_μ_| ϕ_ν_⟩ enters the equation of motion (see SI 4 for details) as follows

$$\frac{\partial \rho }{\partial t}=\frac{-i}{\hbar }\left(S^{-1}H^{\mathrm{eff}}\left(t\right)\rho \left(t\right)-\rho \left(t\right)H^{\mathrm{eff}}\left(t\right)S^{-1}\right)$$ 7

where ***H*** ^eff^ is the matrix with elements of the respective operators introduced in eq 2,

$$H_{\mu \nu }^{\mathrm{eff}}\left(t\right)=\left\langle \phi_{\mu }\right|{Ĥ}^{\mathrm{eff}}\left(t\right)\left|\phi_{\nu }\right\rangle =h_{\mu \nu }^{G_{0}W_{0}}+U_{\mu \nu }\left(t\right)+V_{\mu \nu }^{H}\left[\rho \left(t\right)-\rho \left(0\right)\right]+\Sigma_{\mu \nu }^{\mathrm{SEX}}\left[\rho \left(t\right)-\rho \left(0\right)\right]$$ 8

and **ρ**(*t*) is the time-dependent density matrix with elements ρ_*μν*_(*t*) that satisfy

$$\mathrm{\rho ̂}\left(t\right)=\underset{\mu ,\nu }{\sum }\rho_{\mu \nu }\left(t\right)\left|\phi_{\mu }\right\rangle \left\langle \phi_{\nu }\right|$$ 9

These definitions ensure that time-dependent observables *X*(*t*) of an operator *X̂* have expectation values represented by traces without the presence of the overlap matrix, i.e.

X(t)=Tr(ρ̂(t)X̂)=Tr(ρ(t)X) 10

where ***X*** has matrix elements *X*_*μν*_ = ⟨ϕ_μ_| *X̂* |ϕ_ν_⟩ as discussed in more detail in the SI 4. We note that the presence of ***S*** ^–1^ in (eq 7) can potentially cause problems for very diffuse basis sets for which the matrix inverse becomes ill-conditioned. One strategy to stabilize the inverse is to use basis sets which are optimized for both fast convergence of the excitation energies and small condition number of the overlap matrix, as is done for example in ref 76.

An initial density at time *t* is propagated using the enforced time reversal symmetry (ETRS) scheme^77^

$$\rho \left(t+\Delta t\right)=\exp(-\frac{i}{\hbar }S^{-1}H^{\mathrm{eff}}\left(t+\Delta t\right)\frac{\Delta t}{2})\;\;\rho \left(t+\frac{\Delta t}{2}\right)\exp\left(\frac{i}{\hbar }H^{\mathrm{eff}}\left(t+\Delta t\right)S^{-1}\frac{\Delta t}{2}\right)$$ 11

where

$$\rho \left(t+\frac{\Delta t}{2}\right)=\exp(-\frac{i}{\hbar }S^{-1}H^{\mathrm{eff}}\left(t\right)\frac{\Delta t}{2})\;\;\rho \left(t\right)\exp\left(\frac{i}{\hbar }H^{\mathrm{eff}}\left(t\right)S^{-1}\frac{\Delta t}{2}\right)$$ 12

The matrix exponential in the equations above can be determined using the Baker-Campbell-Hausdorff^77,78^ formula or by exact diagonalization, more information is given in SI 5.

As can be seen from ([Disp-formula eq11]), ETRS is an implicit scheme: ***H*** ^eff^(*t* + Δ*t*) depends on **ρ**(*t* + Δ*t*) that we aim to compute. In our implementation, the implicit dependence is solved by self-consistent (SC) iterations of the density matrix. Specifically, we iterate ***H***_*n*_^eff^(*t* + Δ*t*), where *n* is the iteration number, based on the density matrix ρ_*n*_ as follows

1. ***H***_*n*_^eff^(*t* + Δ*t*) = ***H*** ^eff^[**ρ**_*n*–1_]­(*t* + Δ*t*)
2. **ρ**_*n*_(*t* + Δ*t*) is evaluated from ([Disp-formula eq11])
3. If ||**ρ**_*n*_ – **ρ**_*n*–1_||_max_ < ϵ, cycle is converged

where ||**ρ**||_max_ stands for the element of **ρ** with largest absolute value and ϵ is the SC threshold. For the initial value, we choose $\rho_{0}=\rho \left(t+\frac{\Delta t}{2}\right)$ , determined from ([Disp-formula eq12]). Importantly, the ETRS scheme conserves the idempotency of the density matrix; see SI 6 for more details.

The numerically most expensive part of the ETRS loop is to recalculate the effective Hamiltonian, specifically to compute the SEX self-energy and Hartree matrix. In Gaussian orbitals, these are determined as (see SI 7 for details)

$$V_{\mu \nu }^{H}\left[\rho \left(t\right)\right]=\underset{\lambda ,\sigma }{\sum }\left(\mu \nu |\sigma \lambda \right)\rho_{\lambda \sigma }\left(t\right)$$ 13

where

$$\left(\mu \nu |\sigma \lambda \right)=\int d^{3}rd^{3}r^{\prime }\phi_{\mu }^{*}\left(r\right)\phi_{\nu }\left(r\right)\frac{e^{2}}{4\pi \epsilon_{0}\left|r-r^{\prime }\right|}\phi_{\sigma }^{*}\left(r^{\prime }\right)\phi_{\lambda }\left(r^{\prime }\right)$$ 14

and

$$\Sigma_{\mu \nu }^{\mathrm{SEX}}\left[\rho \left(t\right)\right]=-\underset{\lambda ,\sigma }{\sum }W_{\mu \lambda ,\sigma \nu }\rho_{\lambda \sigma }\left(t\right)$$ 15

where

$$W_{\mu \lambda ,\sigma \nu }=\int d^{3}rd^{3}r^{\prime }\phi_{\mu }^{*}\left(r\right)\phi_{\lambda }\left(r\right)W\left(r,r^{\prime },\omega =0\right)\phi_{\sigma }^{*}\left(r^{\prime }\right)\phi_{\nu }\left(r^{\prime }\right)$$ 16

and *W*(***r***, ***r*** *′*, ω = 0) is the static screened Coulomb interaction, determined from *G*_0_ *W*_0_. In both eq 13 and eq 15, we employ the resolution of identity (RI) to separate the 4-center kernels into 3-center ones.^79,80^ Details are given in SI 8.

Note that the RT-BSE framework discussed in this work is closely related to real-time Hartree–Fock (RT-HF), which forms a fundamental component of real-time TDDFT when hybrid functionals are employed. In RT-HF, the unscreened Coulomb interaction *V* is used in the exchange self-energy, in contrast to the screened Coulomb interaction *W* utilized in the screened-exchange self-energy ([Disp-formula eq15]). Importantly, *W* needs to be computed only once at the beginning of the simulation. As a result, the computational cost of the time propagation is the same in RT-BSE, RT-HF, and real-time TDDFT with hybrid functionals.

The main observable recovered from the time propagation shown in this work is the time-dependent dipole moment

μ(t)=Tr(ρ̂(t)μ̂)=−eTr(ρ̂(t)r̂) 17

The absorption spectrum of the molecules is proportional to the isotropic part of the electric polarizability^81^ α­(ω)

$$\alpha_{jk}\left(\omega \right)=\frac{\mu_{j}\left(\omega \right)}{E_{k}\left(\omega \right)}$$ 18

where μ_*j*_(ω) is the Fourier transform of the component *j* of **μ**(*t*) and *E*_*k*_(ω) is the k-component of the Fourier transform of electric field ***E*** (*t*). The isotropic part is then recovered by a trace

$$\alpha^{\mathrm{iso}}\left(\omega \right)=\frac{1}{3}\mathrm{Tr}\left(\alpha \left(\omega \right)\right)=\frac{1}{3}\underset{j\in \left\{x,y,z\right\}}{\sum }\alpha_{jj}\left(\omega \right)$$ 19

The polarizability determined by the RT-BSE method is compared with the LR-BSE polarizability, which is determined from^17,26^

$$\alpha^{\mathrm{iso}}\left(\omega \right)=\frac{1}{3}\underset{n,j\in \left\{x,y,z\right\}}{\sum }\frac{2\Omega_{n}\left|\left\langle \Psi_{n}\right|{\mathrm{r̂}}_{j}\right|\Psi_{0}\rangle {|}^{2}}{\Omega_{n}^{2}-{\left(\hbar \omega +i\eta \right)}^{2}}$$ 20

where *n* enumerates LR-BSE excitation energies Ω_*n*_, *r̂*_*j*_ is the position operator for position component *j*, η is small artificial broadening, |Ψ_0_⟩ is the equilibrium state and |Ψ_*n*_⟩ is the excitation state.

## 4 Computational Details

### 4.1 Program Parameters

The starting DFT calculation was carried out in the CP2K suite, using the PBE0 ^82,83^ functional with the aug-cc-pVDZ basis set^84–88^ as orbital basis set and aug-cc-pVTZ-RIFIT basis set^89^ as auxiliary basis set for the RI (see SI 9 for RI basis set convergence details). The self-consistent iterations were converged so that the change in density matrix elements between self-consistent field (SCF) iterations was less than 1.0 × 10^–7^ at.u.. The energy cutoff for the plane-wave grid of the density matrix was 600 Ry.

The consecutive low-scaling *G*_0_ *W*_0_ calculation^79^ uses a minimax grid^90–92^ with 30 points in imaginary time and imaginary frequency and a truncation radius of 7 Å in the RI with the truncated Coulomb metric (see SI 10 for effect of the cutoff radius).

The real-time propagation employs the ETRS self-consistent loop^77^ with convergence criterion on the maximum absolute value difference of elements of two consecutive candidate density matrices in atomic orbital basis being less than 10^–7^ atomic units (at.u.). For the presented results, we calculate the matrix exponential using the BCH scheme with threshold 10^–14^ at.u. for the resulting elements of the transformed density matrix.

### 4.2 Fourier Transform and Padé Approximant Refinement

To make meaningful comparisons between the chosen molecules in both LR-BSE and RT-BSE, we identified clear peaks present in both of the refined spectra obtained from the methods, fitted the peaks with Lorentzian function^17^ and compared the peak positions between the two methods.

The initial spectra are obtained by a fast Fourier transform of the time trajectory of the dipole moment **μ**(*t*) of each molecule. We propagate the dynamics for 20 fs, using 20 000 steps of 1 as (time step of 1 as is shown to be converged for cysteine molecule in SI 11, dependence of spectra on total time propagation is illustrated in SI 12). Since the value of the dipole moment at the end is different from the initial value, we apply artificial exponential damping γ ^70,71^ during postprocessing,

$$\mu \left(\omega \right)=\int_{0}^{\infty }dte^{i\left(\omega +i\gamma \right)t}\mu \left(t\right)$$ 21

which acts as a window function of the Fourier transform and effectively broadens the spectrum. A value of γ = 0.2 fs^–1^ was chosen for the damping in order to reduce the amplitude of dipole moment oscillations by e^–4^ by the end of 20 fs of propagation. Further details are given in SI 3.

We obtain refined spectra by fitting a Padé approximant to **μ**(ω) and interpolating on a grid of energies from 0 to 20 eV, with 20 meV steps. The refinement uses *N*/2 Padé parameters, where *N* is the total number of the points considered for the fit, consistently with previous work.^72^ Points of energy up to 200 eV from the initial spectra are used for the fitting. Comparison of spectra with and without Padé interpolation is available in SI 12.

## 5 Accuracy with Respect to LR-BSE

In Figure 1 (a) and (b)­we show that the RT-BSE and LR-BSE are indistinguishable by eye for energies up to 8 eV. For higher energies of formaldehyde in Figure 1 (a), peak positions between LR-BSE and RT-BSE differ by up to ∼0.2 eV. We attribute the increasing deviation for peaks at higher energies to the analytic continuation used in low-scaling *GW* ^79^ which serves as input for RT-BSE to guarantee the same effective computational *N* ^2^ scaling as in RT-BSE. Low-scaling *GW* ^79^ gets inaccurate for quasiparticle energies away from HOMO and LUMO (for *N* ^6^-scaling LR-BSE, we use standard-scaling *GW* ^93^ with 300 frequency points, where the accuracy of analytic continuation is guaranteed in a larger energy window below HOMO and above LUMO). For the specific case of formaldehyde, the HOMO-1 orbital differs in energy by about 0.2 eV between the *GW* implementations, see SI 13 for more details.

**Figure 1:**
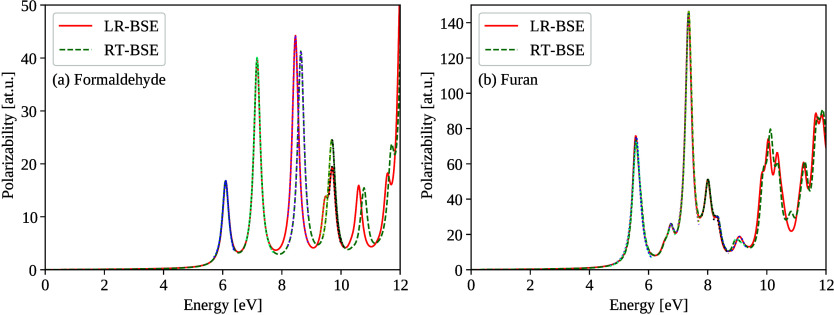
Isotropic polarizability (eq 19) of (a) formaldehyde and (b) furan computed from RT-BSE propagation with Padé interpolation, compared with the LR-BSE approach (eq 20). The blue and yellow dotted lines represent Lorentzian fits used to determine peak positions.

A systematic comparison of the peak positions computed from RT-BSE and LR-BSE across all molecules in Thiel’s set is presented in Figure 2. In Figure 2 (a) we observe that the peaks of RT-BSE and LR-BSE are in excellent agreement; the average absolute deviation is 30 meV, and the largest deviation is around 200 meV. Figure 2 (a) shows the peak position independent of the peak height. Figure 2 (b) shows the absolute difference of peak positions between RT-BSE and LR-BSE, with the color bar indicating the peaks’ relative intensity. For peaks exceeding 50% relative peak amplitude, the average absolute deviation is reduced to 20 meV. Moreover, from Figure 2 (b), formaldehyde can be identified as the outlier regarding numerical precision of RT-BSE, showing the largest deviation of 200 meV between RT-BSE and LR-BSE for peaks with > 50% relative peak intensity. Nevertheless, as seen in Figure 1 (b), the RT-BSE and LR-BSE spectra are indistinguishable by eye up to 8 eV, underscoring the numerical precision of our RT-BSE implementation.

**Figure 2:**
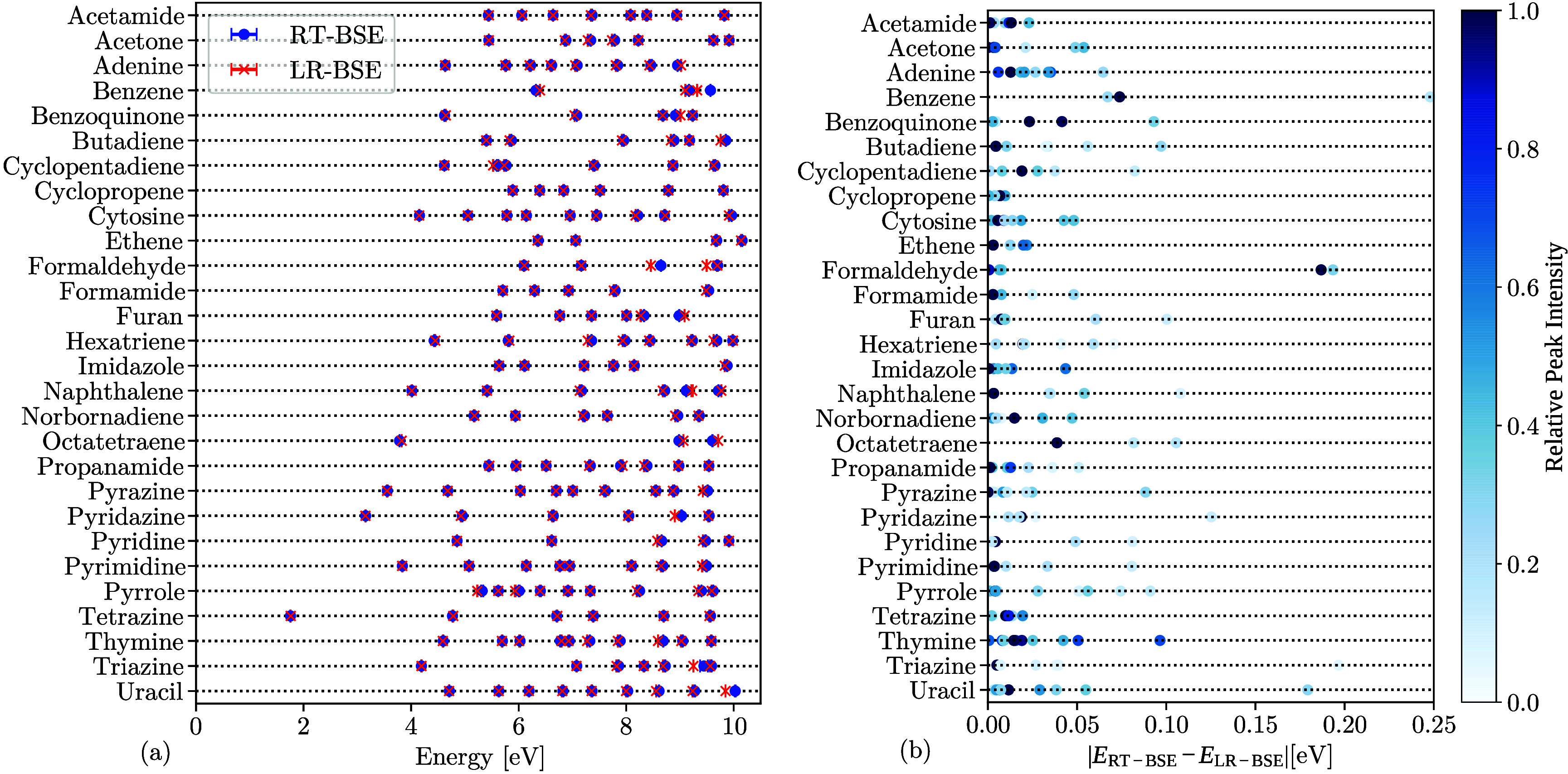
(a) Peak positions obtained from fits to LR-BSE and RT-BSE spectra. We observe excellent agreement in general. (b) Difference between RT-BSE and LR-BSE peak positions, displaying also the relative peak intensity as color—more pronounced peaks in the spectrum (below 10 eV) have more pronounced color. The peaks that differ the most between RT-BSE and LR-BSE (>0.15 eV difference) have usually low relative intensity; i.e., they are not the dominant feature of the molecular spectrum.

## 6 Nonlinear-Optical Effects in Cysteine

In linear optics, the response **μ**(*t*) of the molecule to light is proportional to the electric field amplitude *E*_0_ of the incoming light wave. In nonlinear optics, the response **μ**(*t*) includes higher-order terms of *E*_0_ that become significant for a large *E*_0_. Our chosen molecule to study nonlinear effects is cysteine, an amino acid; see molecular structure sketched in the inset of Figure 3. Cysteine lacks inversion symmetry and therefore allows for second harmonic generation.^40^ Furthermore, being an amino acid, our choice of cysteine demonstrates the applicability of our method to a molecule present in proteins. The linear absorption spectrum of cysteine is shown in Figure 4 with an absorption peak at 6.0 eV. We study nonlinear effects in the electric field amplitude using an electric-field pulse

$$E\left(t\right)=E_{0}\cos\left(\omega_{0}t\right)e^{-{\left(t-t_{0}\right)}^{2}/\left(2\sigma^{2}\right)}$$ 22

where *t*_0_ = 12.6 fs, σ = 4.2 fs, *ℏω*_0_ = 6.0 eV and the field amplitude *E*_0_ was varied between different pulses. We use a simulation time of 52.6 fs in order to access the oscillations without the effect of the pulse field. For the Fourier transform from time to frequency, we use a Gaussian window function (see SI 3.3 for details) centered at 32.6 fs with spread of 4.0 fs. The time trace of the field, *E*(*t*) and dipole moment μ_*x*_(*t*) computed from RT-BSE (using eq 17) are shown in Figure 3.

**Figure 3:**
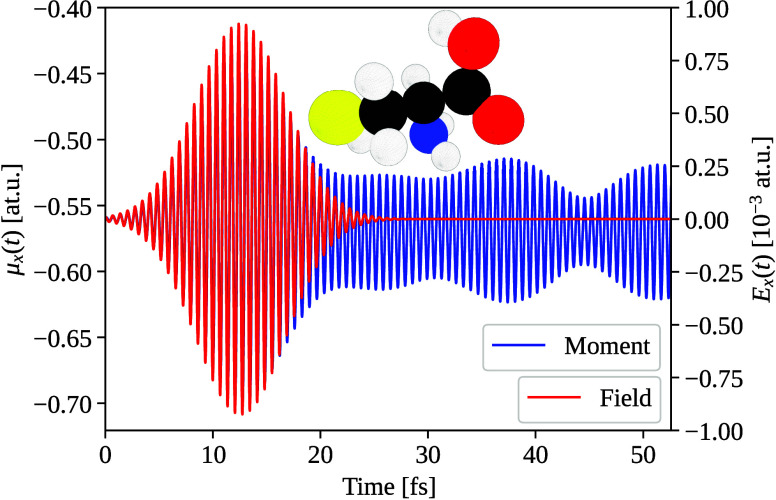
Real-time pulse (in red) and induced dipole moment oscillations (in blue) of the cysteine molecule (molecular geometry shown), along the Cartesian *x* direction. Oscillations of the amplitude after the initial pulse hint at the presence of multiple frequencies. Furthermore, the center of the oscillations after the pulse is shifted, implying average static polarization of the molecule.

**Figure 4:**
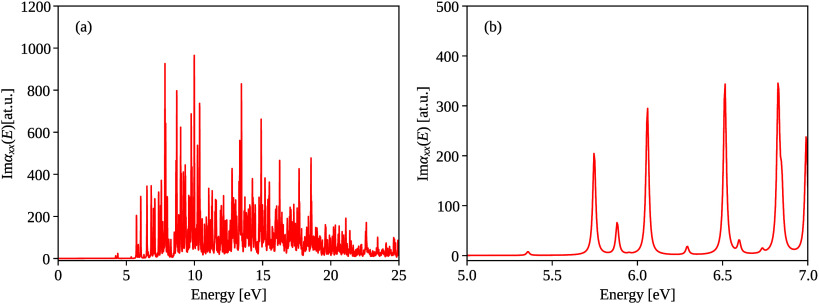
LR-BSE polarizability spectra (a) of cysteine shows many optically active excited states. When focusing on a narrow range around 6.0 eV (shown in (b)), several peaks are within the 1 eV range of 6.0 eV, and hence, several modes are excited by the applied pulse.

Resulting energy-dependent moment magnitudes are shown in Figure 5. We identify the linear response peak in Figure 5 (a) at ∼6 eV and the second harmonic peak at ∼12 eV. Our pulse has a nonzero spectra width ∼1 eV, so several resonance energies Ω_*n*_ get excited, see Figure 4 and (eq 18). Each linear-response peak gets broadened by the window function, so that only a single, broadened linear-response peak at ∼6 eV is visible. For the second-order response at ∼12 eV, we observe two distinct peaks. This is because the spacing of the second-order response peaks is doubled compared with the linear response peaks.

**Figure 5:**
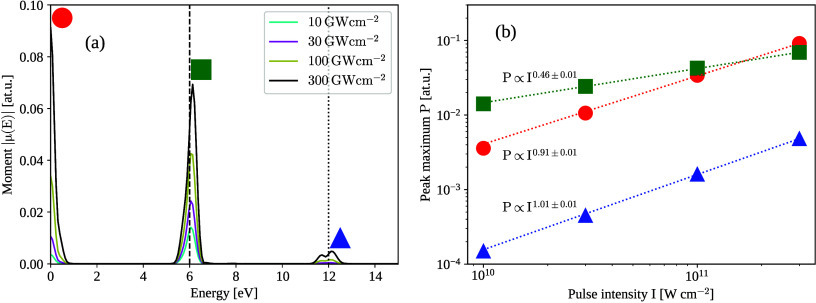
Magnitude of Fourier transform of moment oscillations (a) for various intensities of the applied pulse. The pulse is centered around 6.00 eV (black dashed line). The second harmonic peak around 12.0 eV (black dotted line) emerges with increasing intensity of the pulse. Note that there is also a substantial static shift (optical rectification) of the dipole moment, which also increases with the pulse intensity. The maximum of the moment peaks is shown as a function of the applied intensity on the right (b). We see that the second harmonic peaks and optical rectification scale quadratically with the applied field (linearly with the intensity) and the linear peak scales linearly with the applied field (as square root of the intensity).

The maximum of the second harmonic peak scales linearly with the intensity of the applied pulse *I* = ϵ_0_|*E*_0_|^2^, while the linear response peak maximum scales with $\sqrt{I}$ . Both of these observations are in agreement with the textbook knowledge about linear and nonlinear optics.^40^ We also identified an increasing peak at the zero frequency. Since the maximum of this peak shows the same scaling as the second harmonic peak (see inset of Figure 5), we attribute this peak to optical rectification phenomenon—a second order effect.^40,94^ While we do not expect this effect to be experimentally relevant for molecules (static signal is not optically visible), it might be observable in bulk materials as shift current.^95^ Finally, we note that the shape of the peaks in nonlinear response is influenced by the shape of the pulse, with wider real-time pulse resulting in narrower Fourier transformed peaks. Summarizing, the study of nonlinear optical properties shown here shows the capabilities of RT-BSE applied to organic molecules.

## 7 Conclusions

We have implemented the real-time Bethe–Salpeter equation approach via propagation of the density matrix with screened-exchange self-energy in CP2K. We benchmarked the accuracy of the implementation with respect to the linear response Bethe–Salpeter equation approach, showing an mean average absolute deviation of peak positions in the isotropic polarizability of only 30 meV. The application potential of the method was demonstrated by showing the emergence of the second harmonic peak and optical rectification in the dipole moment magnitude of the amino acid cysteine. The RT-BSE method is an important alternative approach to LR-BSE, expanding it to capture nonlinear optical effects and allowing for explicit control of the pulse shape, both of which are inaccessible in standard LR-BSE.

## Supplementary Material



## Data Availability

Code described in this work is part of the free and open source CP2K suite (*J. Chem. Phys.* **2020**, *152*, 194103). The data described in the work are freely available at NOMAD (https://zenodo.org/records/15235246) repository, Github repository (https://github.com/StepanMarek/RTBSEvsLRBSE), and Zenodo repository (https://zenodo.org/records/15235246).

## Notes

- Additional theoretical and methodological explanations (PDF)

Additional theoretical and methodological explanations: LR-BSE and RT-BSE equivalence, Delta kick for homogeneous excitation, Dipole polarizability from dipole moment time series, Working in overlapping basis, Exponentiation methods, Conservation of idempotency, Hartree and COHSEX terms in Gaussian overlapping basis, RI approximation, RI auxiliary basis convergence, RI cutoff radius convergence, Timestep convergence in HHG of cysteine, Comparison of spectra with and without Padé interpolation, Illustration of low-scaling and standard-scaling eigenvalue difference, and Computational scaling (PDF)
