# Supplementary material for: Linear and Nonlinear Optical Properties of Molecules from Real-Time Propagation Based on the Bethe–Salpeter Equation
Source: J Chem Theory Comput. 2025 Sep 25;21(19):9814–22. doi: 10.1021/acs.jctc.5c01246 (PMC12529910; doi:10.1021/acs.jctc.5c01246)
Supplement: Supplementary file 1 [file ct5c01246_si_001.pdf]

Supporting Information to "Linear and Nonlinear Optical Properties of Molecules from Real-Time Propagation based on the Bethe–Salpeter Equation"

Štěpán Marek* and Jan Wilhelm

Regensburg Center for Ultrafast Nanoscopy and Institute of Theoretical Physics, University of Regensburg, Regensburg, Germany, D-93040

E-mail: stepan.marek@physik.uni-regensburg.de

1 LR-BSE and RT-BSE Equivalence

Before continuing to the specifics of the implementation, let us quickly show the equivalence of the RT-BSE approach with the LR-BSE method in the linear regime. In Ref. 1, authors show that the real-time propagation of density matrix with COHSEX self-energy in linear regime leads to equations are formally equivalent to standard Bethe-Salpeter equation. Here, we briefly show a slightly less general way of deriving the Bethe-Salpeter equation in the explicit form shown for example in Ref. 2, again based on the COHSEX equation of motion (1) in the main text.

Suppose that in equilibrium, the density matrix describing the effective single-particle

states is $\hat{\rho}^0$

$$\hat{\rho}^0 = \sum_m f_m \left| m \right\rangle \left\langle m \right| \ , \tag{1}$$

where $\left| m \right\rangle$ are the equilibrium Hamiltonian ($\hat{H}^0$) eigenstates

$$\hat{H}^0 \left| m \right\rangle = \hbar\omega_m \left| m \right\rangle \ , \tag{2}$$

where $\hbar\omega_m$ is the energy eigenvalue, the eigenvectors are orthonormal $\left\langle m \right| \left. n \right\rangle = \delta_{mn}$ and f_m is a limit of the Fermi distribution for zero temperature - if the highest occupied molecular orbital of the system is M, then

$$f_m = \begin{cases} 1 \text{ if } m \leq M \\ 0 \text{ if } m > M \end{cases} \ . \tag{3}$$

Now, suppose that the state of the system is changed so that the state is now described by density matrix $\hat{\rho}(t) = \hat{\rho}^0 + \Delta\hat{\rho}(t)$. Inserting into the equation of motion leads to

$$\frac{\partial \Delta\hat{\rho}}{\partial t} = \frac{\partial \hat{\rho}}{\partial t} = \frac{-i}{\hbar} \left[\hat{H}^{\text{eff}}(t), \hat{\rho}^0 + \Delta\hat{\rho}(t) \right] \ . \tag{4}$$

Without the external field $\hat{U}(t)$, the only time-dependence in the Hamiltonian is present due to the changes in the density matrix, i.e.

$$\begin{aligned} \frac{\partial \Delta\hat{\rho}}{\partial t} = \frac{-i}{\hbar} \Big(\left[\hat{H}^0, \hat{\rho}^0 \right] + \left[\hat{H}^0, \Delta\hat{\rho}(t) \right] + \left[\hat{V}^{\text{Hartree}}[\hat{\rho}(t)] - \hat{V}^{\text{Hartree}}[\hat{\rho}^0], \hat{\rho}^0 + \Delta\hat{\rho}(t) \right] + \\ + \left[\hat{\Sigma}^{\text{COHSEX}}[\hat{\rho}(t)] - \hat{\Sigma}^{\text{COHSEX}}[\hat{\rho}^0], \hat{\rho}^0 + \Delta\hat{\rho}(t) \right] \Big) \ . \end{aligned} \tag{5}$$

The first commutator is zero. For the resolution of the rest, it is useful to transform to

the basis of eigenstates of $\hat{H}^0$

$$\frac{\partial \Delta\rho_{mn}}{\partial t} = -\frac{i}{\hbar}\Bigg(\left[\boldsymbol{H}^0, \Delta\boldsymbol{\rho}(t)\right]_{mn} + \\ + \sum_{p,q,r} (mp|rq)\Delta\rho_{qr}(t)(\boldsymbol{\rho}^0 + \Delta\boldsymbol{\rho}(t))_{pn} - (\boldsymbol{\rho}^0 + \Delta\boldsymbol{\rho}(t))_{mp}(pn|rq)\Delta\rho_{qr}(t) - \\ - \sum_{p,q,r} W_{mq,rp}\Delta\rho_{qr}(t)(\boldsymbol{\rho}^0 + \Delta\boldsymbol{\rho}(t))_{pn} - (\boldsymbol{\rho}^0 + \Delta\boldsymbol{\rho}(t))_{mp}W_{pq,rn}\Delta\rho_{qr}(t)\Bigg) \ . \tag{6}$$

Now, we restrict ourselves to linear regime, i.e., we neglect higher than linear powers of $\Delta\boldsymbol{\rho}(t)$. Furthermore, we substitute for $\boldsymbol{\rho}^0$ from (1), which leads to

$$\frac{\partial \Delta\rho_{mn}}{\partial t} = -\frac{i}{\hbar}\Bigg(\hbar(\omega_m - \omega_n)\Delta\rho_{mn}(t) + \\ + \sum_{p,q} (mn|qp)\Delta\rho_{pq}(t) f_n - f_m (mn|qp)\Delta\rho_{pq}(t) - \\ - \sum_{p,q} W_{mp,qn}\Delta\rho_{pq}(t) f_n - f_m W_{mp,qn}\Delta\rho_{pq}(t)\Bigg) \ . \tag{7}$$

After some reordering, the equation of motion in linear response regime for the perturbation $\Delta\boldsymbol{\rho}(t)$ is

$$\frac{\partial \Delta\rho_{mn}}{\partial t} = i(\omega_n - \omega_m)\Delta\rho_{mn}(t) - \frac{i}{\hbar}(f_n - f_m)\sum_{p,q}\left((mn|qp) - W_{mp,qn}\right)\Delta\rho_{pq}(t) \tag{8}$$

By applying the Fourier transform, we obtain

$$\hbar\omega\Delta\rho_{mn}(\omega) = -\hbar(\omega_n - \omega_m)\Delta\rho_{mn}(E) + (f_n - f_m)\sum_{p,q}\left((mn|qp) - W_{mp,qn}\right)\Delta\rho_{pq}(\omega) \tag{9}$$

1.1 Linear Response Density via Delta-Kick

Consider now that the initial non-equilibrium part of the density results from applying the delta-kick, described later on. The effect of the delta kick can be described as

$$\hat{\rho}(t=0^+) = \mathrm{e}^{-i\hat{A}}\hat{\rho}^0\mathrm{e}^{i\hat{A}} = \hat{\rho}^0 + \Delta\rho(t=0)\,, \tag{10}$$

where $\hat{A}$ is a Hermitian operator. Evaluating $\hat{\rho}(t=0^+)$ in the basis of original Hamiltonian eigenstates $|m\rangle$ leads to

$$\langle m|\,\hat{\rho}(t=0^+)\,|n\rangle = \sum_{p,q} \langle m|\,\mathrm{e}^{-i\hat{A}}\,|p\rangle\,\langle p|\,\hat{\rho}^0\,|q\rangle\,\langle q|\,\mathrm{e}^{i\hat{A}}\,|n\rangle \tag{11}$$

Approximating the exponential and resulting matrix products to linear order in $\hat{A}$ leads to

$$\langle m|\,\hat{\rho}(t=0^+)\,|n\rangle = \langle m|\,\hat{\rho}^0\,|n\rangle + \sum_{p,q}(-i)\,\langle m|\,\hat{A}\,|p\rangle\,\langle p|\,\hat{\rho}^0\,|q\rangle\,\delta_{qn} + i\delta_{mp}\,\langle p|\,\hat{\rho}^0\,|q\rangle\,\langle q|\,\hat{A}\,|n\rangle \tag{12}$$

Substituting from (1)

$$\langle m|\,\hat{\rho}(t=0^+)\,|n\rangle = f_m\delta_{mn} + \sum_{p}(-i)A_{mp}f_p\delta_{pn} + if_m\delta_{mq}A_{qn} = f_m\delta_{mn} + i(f_m - f_n)A_{mn} \tag{13}$$

First of all, we note that the perturbed density matrix remains Hermitian

$$\begin{aligned}\langle n|\,\hat{\rho}(t=0^+)\,|m\rangle^* &= (f_n\delta_{nm} + i(f_n - f_m)A_{nm})^* = \\ &= f_m\delta_{mn} + i(f_m - f_n)A_{mn} = \langle m|\,\hat{\rho}(t=0^+)\,|n\rangle\end{aligned} \tag{14}$$

since $\hat{A}$ is Hermitian. The $f_m\delta_{mn}$ term corresponds to equilibrium density element $\langle m|\,\hat{\rho}^0\,|n\rangle$. The second term (corresponding to $\Delta\rho(0)$) is non-zero only if one of m, n belongs to the occupied subspace while the other belongs to the unoccupied subspace. We can change the

second term further

$$\langle m|\,\Delta\hat{\rho}(0)\,|n\rangle = iA_{mn}(f_m - f_n) = iA_{mn}(f_m - f_m f_n + f_m f_n - f_n) =$$
$$= iA_{mn}f_m(1-f_n) - iA_{mn}f_n(1-f_m) \tag{15}$$

To summarize, the density matrix perturbation acquires the off-diagonal occupied/unoccupied block structure as a response to weak (linear response regime) unitary transformation of the density matrix away from the equilibrium.

1.2 Casida Formalism

Substituting to (9) from (15) and defining $X_{mn} = iA_{mn}$ and $Y_{mn} = -iA_{nm}$, we get

$$\begin{aligned}
&\hbar\omega(X_{mn}(\omega)f_m(1-f_n) + Y_{nm}(\omega)f_n(1-f_m)) = \\
&-\hbar(\omega_n - \omega_m)(X_{mn}(\omega)f_m(1-f_n) + Y_{nm}(\omega)f_n(1-f_m)) + \\
&+(f_n - f_m)\sum_{p,q}((mn|qp) - W_{mp,qn})(X_{pq}(\omega)f_p(1-f_q) + Y_{qp}(\omega)f_q(1-f_p))
\end{aligned} \tag{16}$$

We now notice that only the values of m, n for occupied + unoccupied orbital pairs lead to non-trivial equations. Specifically, setting i, j to stand for indices of occupied orbitals and a, b for indices of unoccupied orbitals, we have a set of two coupled equations

$$\hbar\omega X_{ia}(\omega) = \hbar(\omega_a - \omega_i)X_{ia}(\omega) - \sum_{j\text{ occ.},b\text{ unocc.}}((ia|bj) - W_{ij,ba})X_{jb} + ((ia|jb) - W_{ib,ja})Y_{jb} \tag{17}$$

$$\hbar\omega Y_{ia}(E) = -\hbar(\omega_a - \omega_i)Y_{ia}(E) + \sum_{j\text{ occ.},b\text{ unocc.}}((ai|bj) - W_{aj,bi})Y_{jb} + ((ai|jb) - W_{ab,ji})X_{jb} \tag{18}$$

This is equivalent to the Bethe-Salpeter approach via the Casida equation. Specifically,

defining

$$A_{ia,jb} = \hbar(\omega_a - \omega_i)\delta_{ij}\delta_{ab} - (ia|bj) + W_{ij,ba}$$
$$B_{ia,jb} = -(ia|jb) + W_{ib,ja}\ , \tag{19}$$

one can write

$$\begin{pmatrix} \boldsymbol{A} & \boldsymbol{B} \\ -\boldsymbol{B}^\dagger & -\boldsymbol{A}^\dagger \end{pmatrix} \begin{pmatrix} \boldsymbol{X} \\ \boldsymbol{Y} \end{pmatrix} = \hbar\omega \begin{pmatrix} \boldsymbol{X} \\ \boldsymbol{Y} \end{pmatrix} \tag{20}$$

which is consistent for example with notation in Liu et al.[2] or in Sander and Kresse.[3]

2 Delta Kick for Homogeneous Excitation

Instead of a time resolved field pulse $\boldsymbol{E}(t)$, we might choose to apply a delta-like pulse

$$\boldsymbol{E}(t) = I\boldsymbol{\epsilon}\delta(t) \tag{21}$$

where I is the scale of the delta pulse and $\boldsymbol{\epsilon}$ is the polarization.[4] We resolve the effect of the delta pulse on the density of states via explicitly integrating the equation of motion. Let $\hat{\rho}^-$ be the density matrix operator before the application of the pulse and $\hat{\rho}^+$ the density matrix operator immediately after the application, i.e.

$$\hat{\rho}^{\pm} = \lim_{\Delta t \to 0^+} \hat{\rho}(\pm\frac{1}{2}\Delta t) \tag{22}$$

Before the application of the pulse, $\hat{\rho}^-$ corresponds to the starting $\hat{\rho}_0$ provided by the initial guess from DFT+G_0W_0. The equation of motion to zeroth order in I is solved as

$$\hat{\rho}^+ = \hat{\rho}^- - \frac{i}{\hbar}\int_{-\Delta t/2}^{\Delta t/2} dt[\hat{H}_0, \hat{\rho}(t)] \approx \hat{\rho}^- - \frac{i}{\hbar}\Delta t[\hat{H}_0, \hat{\rho}^-] \tag{23}$$

where $\hat{H}_0$ is the time-independent DFT+G_0W_0 Hamiltonian. To the first order in I

$$\hat{\rho}^+ = \hat{\rho}^- - \frac{i}{\hbar}\int_{-\Delta t/2}^{\Delta t/2} dt[\hat{H}_0 + I\delta(t)\boldsymbol{\epsilon}\cdot\hat{r}, \hat{\rho}(t)] \approx \tag{24}$$

$$\approx \hat{\rho}^- - \frac{i}{\hbar}\int_{-\Delta t/2}^{\Delta t/2} dt\left[\hat{H}_0 + I\delta(t)\boldsymbol{\epsilon}\cdot\hat{r}, \hat{\rho}^- - \frac{i}{\hbar}\int_{-\Delta t/2}^{t} dt'[\hat{H}_0 + I\delta(t')\boldsymbol{\epsilon}\cdot\hat{r}, \hat{\rho}^-]\right] = \tag{25}$$

$$= \hat{\rho}^- - \frac{i}{\hbar}[\hat{H}_0, \hat{\rho}^-]\Delta t + \left(\frac{-i}{\hbar}\right)^2 [\hat{H}_0, [\hat{H}_0, \hat{\rho}^-]]\underbrace{\int_{-\Delta t/2}^{\Delta t/2} dt \int_{-\Delta t/2}^{\Delta t/2} dt'}_{(\Delta t)^2/2} + \tag{26}$$

$$+\left(\frac{-i}{\hbar}\right)^2 [\hat{H}_0, [I\boldsymbol{\epsilon}\cdot\hat{r}, \hat{\rho}^-]]\Delta t - \frac{i}{\hbar}[I\boldsymbol{\epsilon}\cdot\hat{r}, \hat{\rho}^-] + \tag{27}$$

$$+\left(\frac{-i}{\hbar}\right)^2 [I\boldsymbol{\epsilon}\cdot\hat{r}, [\hat{H}_0, \hat{\rho}^-]]\underbrace{\int_{-\Delta t/2}^{\Delta t/2} dt\delta(t) \int_{-\Delta t/2}^{t} dt' 1}_{\Delta t/2} \tag{28}$$

Now, we can notice that only the terms ρ^- and $(-i)/\hbar[I\boldsymbol{\epsilon}\cdot\hat{r}, \hat{\rho}^-]$ are independent of Δt and will therefore be finite even for $\Delta t \to 0$.

Similarly, we derive that only term independent of Δt to the second order in I will be

$$\left(\frac{-i}{\hbar}\right)^2 [I\boldsymbol{\epsilon}\cdot\hat{r}, [I\boldsymbol{\epsilon}\cdot\hat{r}, \hat{\rho}^-]]\int_{-\Delta t/2}^{\Delta t/2} dt\delta(t) \int_{-\Delta t/2}^{t} dt'\delta(t') = \tag{29}$$

$$= \left(\frac{-i}{\hbar}\right)^2 [I\boldsymbol{\epsilon}\cdot\hat{r}, [I\boldsymbol{\epsilon}\cdot\hat{r}, \hat{\rho}^-]]\int_{-\Delta t/2}^{\Delta t/2} dt\delta(t)\theta(t) = \frac{1}{2}\left(\frac{-i}{\hbar}\right)^2 [I\boldsymbol{\epsilon}\cdot\hat{r}, [I\boldsymbol{\epsilon}\cdot\hat{r}, \hat{\rho}^-]] \tag{30}$$

and equivalently for higher orders, leading to expression replicating the BCH theorem[5]

$$\hat{\rho}^+ = \sum_{n=0}^{\infty}\frac{1}{n!}\left[\left(\frac{-i}{\hbar}\right) I\boldsymbol{\epsilon}\cdot\hat{r}, \hat{\rho}^-\right]_n = e^{-\frac{i}{\hbar}I\boldsymbol{\epsilon}\cdot\hat{r}}\hat{\rho}^- e^{\frac{i}{\hbar}I\boldsymbol{\epsilon}\cdot\hat{r}} \tag{31}$$

The expression in atomic basis follows exactly the BCH/exact diagonalisation scheme discussed later, with substitution of $\boldsymbol{H}(t)$ by $I\boldsymbol{\epsilon}\cdot\hat{r}$.

3 (Dipole) Polarizability from Dipole Moment Time Series

The dipole polarizability $\alpha_{mn}(\omega)$ ($m, n \in \{x, y, z\}$ are Cartesian directions) is recovered from the time series of the dipole operator $\hat{\mu}_m$ expectation value and the time series of the applied field $E_n(t)$ as

$$\alpha_{mn}(\omega) = \frac{\mu_m(\omega)}{E_n(\omega)} = \frac{\int_{t_0}^{\infty} dt \mathrm{e}^{\mathrm{i}\omega(t-t_0)} \mathrm{e}^{-\gamma(t-t_0)} \langle \hat{\mu}_m(t) - \hat{\mu}_m(t_0) \rangle}{\int_{t_0}^{\infty} dt \mathrm{e}^{\mathrm{i}\omega(t-t_0)} E_n(t)} \tag{32}$$

where t_0 is a reference time before which the density matrix is stationary (before application of the excitation), $\hat{\mu}_m(t)$ is the dipole moment operator for Cartesian direction $m \in \{x, y, z\}$, γ is an artificial damping which stabilizes the numerical Fourier transform[6] and $E_n(t)$ is the external field along direction n.

3.1 Optical Spectra from Real Time Moment Traces

The absorption is given (quasi-classically) by the acceleration of the dipole moment, which introduces an extra factor of ω. The absorption of the field is associated with decreasing amplitude of the resulting field, i.e. with the imaginary value of α. Specifically,[4,6–10]

$$S(\omega) \propto \frac{1}{3} \omega \mathrm{Tr} \left(\mathrm{Im} \left(\boldsymbol{\alpha}(\omega) \right) \right) \ . \tag{33}$$

3.2 Spectrum Post-Processing

The discrete Fourier transform is used to get the transform of the dipole moment oscillations. In order to reduce numerical noise, several post-processing steps are applied.

Firstly, a damping envelope is applied to the real time signal. For majority of the results, the chosen envelope is the exponential damping, as shown in (32), which leads to effective

broadening of the peaks of the oscillation, as can be seen from FT of

$$\int dte^{i\omega t}\theta(t)\sin(\omega_0 t)e^{-\gamma t} = \int_0^\infty e^{i(\omega+i\gamma)t}\sin(\omega_0 t) = \tag{34}$$

$$= \frac{1}{2i}\int_0^\infty dt\left[e^{i(\omega+\omega_0+i\gamma)t} - e^{i(\omega-\omega_0+i\gamma)t}\right] = \frac{1}{2}\left[\frac{1}{\omega+\omega_0+i\gamma} - \frac{1}{\omega-\omega_0+i\gamma}\right] \tag{35}$$

The sine was chosen as an oscillatory function that has 0 value at $t = 0$. Besides numerical stabilization of the peak positions by broadening, the exponential damping also leads to coinciding initial and final value of the moments, which removes some abnormal frequencies otherwise present in the FT.

The imaginary part of the FT is than the imaginary part of the expression in the brackets above, which results in a Lorentzian peak

$$\frac{1}{2}\left[\frac{-\gamma}{(\omega+\omega_0)^2+\gamma^2} + \frac{\gamma}{(\omega-\omega_0)^2+\gamma^2}\right] \tag{36}$$

The polarizability depends also on the transform of the applied field. Therefore, we set $t = 0$ at the symmetry centre of non-oscillatory envelope of the field - this leads to a purely real Fourier transform. Numerically, this is achieved by wrapping the sample around certain index of the signal data array.

3.3 Other Possible Window Functions

Note that one can also choose symmetric window functions such as Gaussian and symmetric exponential (Poisson) window function. For a general signal $f(t)$ with Fourier transform

$$f(\omega) = \sum_i c_i\delta(\omega-\omega_i)\ , \tag{37}$$

the effect of the window function $h(t)$ is understood through the convolution in Fourier space. Specifically,

$$\int_{-\infty}^{\infty} f(t)h(t)e^{i\omega t}dt = \int_{-\infty}^{\infty} dt \frac{1}{4\pi^2}\int_{-\infty}^{\infty} d\omega \int_{-\infty}^{\infty} d\omega' e^{-i\omega' t}e^{-i\omega'' t}e^{i\omega t}f(\omega')h(\omega'') = \quad (38)$$

$$= \int_{-\infty}^{\infty} d\omega'' \int_{-\infty}^{\infty} d\omega' f(\omega')h(\omega'')\frac{1}{2\pi}\delta(\omega' + \omega'' - \omega) = \quad (39)$$

$$= \frac{1}{2\pi}\int d\omega' f(\omega')h(\omega - \omega') = \quad (40)$$

$$= \frac{1}{2\pi}\sum_i c_i h(\omega - \omega_i)\ , \quad (41)$$

i.e. the Fourier transform of the window function replaces the delta peaks in the spectrum $f(\omega)$ of original signal $f(t)$. For even more specific case of sine signal

$$f(t) = \sum_i a_i \sin(\omega_i t) \quad (42)$$

we have

$$f(\omega) = i\pi \sum_i a_i(\delta(\omega - \omega_i) - \delta(\omega + \omega_i)) \quad (43)$$

For exponential window $h(t) = e^{-\gamma|t|}$, so

$$h(\omega) = \frac{2\gamma}{\omega^2 + \gamma^2} \quad (44)$$

and hence

$$\int_{-\infty}^{\infty} f(t)h(t)e^{i\omega t}dt = i\gamma \sum_i \frac{a_i}{(\omega - \omega_i)^2 + \gamma^2} - \frac{a_i}{(\omega + \omega_i)^2 + \gamma^2} \quad (45)$$

For Gaussian window function $h(t) = e^{-t^2/(2\sigma^2)}$, $h(\omega) = \sigma\sqrt{2\pi}e^{-\sigma^2\omega^2/2}$, so the sine signal

produces

$$\int_{-\infty}^{\infty} f(t)h(t)e^{i\omega t}dt = i\sigma\sqrt{\frac{\pi}{2}}\sum_i a_i e^{-\sigma^2(\omega-\omega_i)^2/2} - a_i e^{-\sigma^2(\omega+\omega_i)^2/2} \tag{46}$$

In summary, both the exponential and the Gaussian window function produce a positive semi-definite imaginary spectrum in the positive frequency range for sine signal.

4 Working in Overlapping Basis - Covariant and Contravariant Operator Representations

In an overlapping basis of state vectors, we have a set of vectors $|\phi_\mu\rangle$ (we shall assume finite) which satisfy

$$\langle\phi_\mu|\,\phi_\nu\rangle = S_{\mu\nu} \tag{47}$$

where $S_{\mu\nu}$ is the so-called overlap matrix. Assume now that a new set of vectors $|\psi_m\rangle$ is constructed as linear combinations of $|\phi_\mu\rangle$,

$$|\psi_m\rangle = \sum_\mu C_{\mu m}\,|\phi_\mu\rangle\,, \langle\psi_m|\,\psi_n\rangle = \delta_{mn}\,, \tag{48}$$

where δ_{mn} is the Kronecker delta. The condition on coefficients $C_{\mu m}$ can be then formulated as matrix equation

$$\boldsymbol{C}^\dagger \boldsymbol{S}\boldsymbol{C} = \boldsymbol{I}\,, \tag{49}$$

where $\boldsymbol{I}$ is the identity matrix, or alternatively

$$\boldsymbol{S}^{-1} = \boldsymbol{C}\boldsymbol{C}^\dagger\,. \tag{50}$$

For representation of quantum mechanical operators as matrices, one usually employs the completeness relation to resolve the matrix elements. In orthogonal basis, this takes form $\sum_m |\psi_m\rangle \langle\psi_m| = \hat{I}$, where $\hat{I}$ is the identity operator. In the overlapping basis, this can be expressed as

$$\sum_m \left(\sum_\mu C_{\mu m} |\phi_\mu\rangle \right) \left(\sum_\nu C^*_{\nu m} \langle\phi_\nu| \right) = \sum_{\mu,\nu} |\phi_\mu\rangle \left(\sum_m C_{\mu m} C^*_{\nu m} \right) \langle\phi_\nu| = \sum_{\mu,\nu} |\phi_\mu\rangle S^{-1}_{\mu\nu} \langle\phi_\nu| \tag{51}$$

We crucially note that the last expression is independent of the choice of the orthonormal basis and therefore represents the identity operator and associated completeness relation in the overlapping basis.

The presence of the overlap matrix in the completeness relation leads to difference in the representation of operators, which we call covariant and contravariant representation. In the covariant representation, we represent the operators by matrices

$$A_{\mu\nu} = \langle\phi_\mu| \hat{A} |\phi_\nu\rangle \ , \tag{52}$$

while in the contravariant representation, we use

$$\hat{A} = \sum_{\mu,\nu} |\phi_\mu\rangle A_{\mu\nu} \langle\phi_\nu| \ . \tag{53}$$

The two representations are related by transformation

$$\boldsymbol{A}_{\mathrm{cov}} = \boldsymbol{S} \boldsymbol{A}_{\mathrm{contra}} \boldsymbol{S} \ . \tag{54}$$

4.1 Reasoning Behind Representation Naming

When the basis is transformed by a unitary transformation $\hat{U}$, such that

$$\left|\phi'_\mu\right\rangle = \hat{U}\left|\phi_\mu\right\rangle \tag{55}$$

the covariant representation of the operator transforms as

$$(\boldsymbol{A}')_{\mu\nu} = \left\langle\phi'_\mu\right|\hat{A}\left|\phi'_\nu\right\rangle = \left\langle\phi_\mu\right|\hat{U}^\dagger\hat{A}\hat{U}\left|\phi_\nu\right\rangle = \boldsymbol{U}^\dagger\boldsymbol{S}^{-1}\boldsymbol{A}\boldsymbol{S}^{-1}\boldsymbol{U} \tag{56}$$

while the contravariant representation transforms as

$$\hat{A} = \sum_{\mu,\nu}\left|\phi'_\mu\right\rangle(\boldsymbol{A}')_{\mu\nu}\left\langle\phi'_\nu\right| = \sum_{\mu,\nu}\hat{U}\left|\phi_\mu\right\rangle(\boldsymbol{A}')_{\mu\nu}\left\langle\phi_\nu\right|\hat{U}^\dagger = \sum_{\mu,\nu}(\boldsymbol{S}^{-1}\boldsymbol{U}\boldsymbol{A}'\boldsymbol{U}^\dagger\boldsymbol{S}^{-1})_{\mu\nu}\left|\phi_\mu\right\rangle\left\langle\phi_\nu\right| \tag{57}$$

which can be rewritten as

$$(\boldsymbol{A}')_{\mu\nu} = (\boldsymbol{S}^{-1}\boldsymbol{U}^\dagger\boldsymbol{A}\boldsymbol{U}\boldsymbol{S}^{-1})_{\mu\nu}\,. \tag{58}$$

Here, we used $\left\langle\phi_\mu\right|\hat{U}^{(\dagger)}\left|\phi_\nu\right\rangle = (\boldsymbol{U}^{(\dagger)})_{\mu\nu}$ and unitarity condition $\boldsymbol{U}\boldsymbol{S}^{-1}\boldsymbol{U}^\dagger = \boldsymbol{S}$.

The covariant representation transforms with $\boldsymbol{S}^{-1}$ directly next to operator components, which is the same as in the completeness relation, while the contravariant representation transforms with $\boldsymbol{U}/\boldsymbol{U}^\dagger$ directly next to components. We note that in an orthonormal basis, overlap matrices become identity matrices, and hence the representations become identical.

4.2 Mixed Operator Representation in CP2K

In CP2K, most operators are represented covariantly, while the density matrix is represented contravariantly. This has an important effect on the numerical calculation of the trace of

operators - in the orthonormal basis, expectation values of operators are often evaluated as

$$\left\langle \hat{A} \right\rangle = \mathrm{Tr}\left(\hat{\rho}\hat{A}\right) = \sum_m \langle\psi_m|\,\hat{\rho}\hat{A}\,|\psi_m\rangle \tag{59}$$

Expressing the orthonormal vectors in terms of the non-orthogonal basis leads to

$$\mathrm{Tr}\left(\hat{\rho}\hat{A}\right) = \sum_{\mu,\nu,m} \langle\phi_\mu|\, C^*_{\mu m}\hat{\rho}\hat{A}C_{\nu m}\,|\phi_\nu\rangle = \sum_{\mu,\nu,\mu',\nu',m} C_{\nu m}C^*_{\mu m}S_{\mu\mu'}\rho_{\mu'\nu'}\,\langle\phi_{\nu'}|\,\hat{A}\,|\phi_\nu\rangle\ , \tag{60}$$

where we substituted for the contravariant representation of $\hat{\rho}$. We now substitute from (50), which leads to

$$\mathrm{Tr}\left(\hat{\rho}\hat{A}\right) = \sum_{\nu,\nu'} \rho_{\nu\nu'}\,\langle\phi_{\nu'}|\,\hat{A}\,|\phi_\nu\rangle\ , \tag{61}$$

meaning that using the covariant representation for $\hat{A}$ will lead to operator trace being calculated as numerical trace of the matrix representation.

4.3 Propagation of Matrices in Overlapping Basis

The equation of motion ((1) in the main text) can be represented in the overlapping Gaussian basis using the contravariant representation for the density operator and the covariant representation for the Hamiltonian operator as

$$\boldsymbol{S}\frac{\partial\boldsymbol{\rho}}{\partial t}\boldsymbol{S} = -\frac{i}{\hbar}\left(\boldsymbol{H}(t)\boldsymbol{\rho}(t)\boldsymbol{S} - \boldsymbol{S}\boldsymbol{\rho}(t)\boldsymbol{H}(t)\right) \tag{62}$$

$$\frac{\partial\boldsymbol{\rho}}{\partial t} = -\frac{i}{\hbar}\left(\boldsymbol{S}^{-1}\boldsymbol{H}(t)\boldsymbol{\rho}(t) - \boldsymbol{\rho}(t)\boldsymbol{H}(t)\boldsymbol{S}^{-1}\right) \tag{63}$$

An approximate solution to this differential equation would predict a density matrix at

time $t + \Delta t$ using density matrix at time t as

$$\boldsymbol{\rho}(t+\Delta t) = e^{-(i/\hbar)\boldsymbol{S}^{-1}\boldsymbol{H}(t)\Delta t}\boldsymbol{\rho}(t)e^{(i/\hbar)\boldsymbol{H}(t)\boldsymbol{S}^{-1}\Delta t} \tag{64}$$

i.e. by neglecting the variation of $\boldsymbol{H}$ via approximation $\forall t' \in [t, t+\Delta t] : \boldsymbol{H}(t') = \boldsymbol{H}(t)$.

The variation of $\boldsymbol{H}$ with time can be included by self-consistent schemes such as the enforced time reversal symmetry scheme (ETRS), as described in the main text.

5 Exponentiation Methods

5.1 Exact Diagonalisation

In the exact diagonalisation scheme, Hamiltonian matrix $\boldsymbol{H}(t)$ is diagonalized at each time t, which results in eigenvectors $\boldsymbol{C}(t)$

$$\boldsymbol{H}(t)\boldsymbol{C}(t) = \boldsymbol{S}\boldsymbol{C}(t)\boldsymbol{\Lambda}(t) \tag{65}$$

where $\boldsymbol{\Lambda}(t)$ is the diagonal matrix of eigenvalues. Using (50)

$$\boldsymbol{H}(t) = \boldsymbol{S}\boldsymbol{C}(t)\boldsymbol{\Lambda}(t)\boldsymbol{C}^{\dagger}(t)\boldsymbol{S} \tag{66}$$

and therefore

$$e^{-(i/\hbar)\Delta t\boldsymbol{S}^{-1}\boldsymbol{H}(t)} = \sum_{n=0}^{\infty}\left(\frac{-i\Delta t}{\hbar}\right)^{n}\frac{1}{n!}(\boldsymbol{C}(t)\boldsymbol{\Lambda}(t)\boldsymbol{C}^{\dagger}(t)\boldsymbol{S})^{n} = \tag{67}$$

$$= \sum_{n=0}^{\infty}\frac{1}{n!}\boldsymbol{C}(t)\left(\frac{-i\Delta t}{\hbar}\boldsymbol{\Lambda}(t)\right)^{n}\boldsymbol{C}^{\dagger}(t)\boldsymbol{S} = \boldsymbol{C}(t)e^{-(i/\hbar)\Delta t\boldsymbol{\Lambda}(t)}\boldsymbol{C}^{\dagger}(t)\boldsymbol{S} \tag{68}$$

where the exponential of diagonal matrix $-(i/\hbar)\Delta t\boldsymbol{\Lambda}(t)$ is trivial to evaluate.

5.2 BCH Scheme

Baker-Campbell-Hausdorff formula[5,11] establishes that for two operators $\hat{A}$ and $\hat{B}$, the product

$$e^{\hat{A}}\hat{B}e^{-\hat{A}} = \sum_{n=0}^{\infty} \frac{1}{n!}[\hat{A}, \hat{B}]_n \tag{69}$$

where

$$[\hat{A}, \hat{B}]_n = [\hat{A}, [\hat{A}, \hat{B}]_{n-1}],\ [\hat{A}, \hat{B}]_0 = \hat{B} \tag{70}$$

In non-orthogonal basis, the commutators are resolved as

$$([\hat{A}, \hat{B}]_n)_{\mu\nu} = (\langle\phi_\mu| \hat{A}[\hat{A}, \hat{B}]_{n-1} |\phi_\nu\rangle - \langle\phi_\mu| [\hat{A}, \hat{B}]_{n-1}\hat{A} |\phi_\nu\rangle) = \tag{71}$$

$$= \sum_{\mu',\nu'} \left(A_{\mu\mu'} S^{-1}_{\mu'\nu'} ([\hat{A}, \hat{B}]_{n-1})_{\nu'\nu} - ([\hat{A}, \hat{B}]_{n-1})_{\mu\mu'} S^{-1}_{\mu'\nu'} A_{\nu'\nu} \right) \tag{72}$$

where

$$([\hat{A}, \hat{B}]_n)_{\mu\nu} = \langle\phi_\mu| [\hat{A}, \hat{B}]_n |\phi_\mu\rangle \tag{73}$$

The mathematics is again simplified if we choose a mixed representation - we shall represent $\hat{A}$ as covariant and the commutators as contravariant, i.e. we define $C_{n,\mu\nu}$

$$[\hat{A}, \hat{B}]_n = \sum_{\mu,\nu} C_{n,\mu\nu} |\phi_\mu\rangle \langle\phi_\nu| \tag{74}$$

$$([\hat{A}, \hat{B}]_n)_{\mu\nu} = (\boldsymbol{S}\boldsymbol{C}_n\boldsymbol{S})_{\mu\nu} \tag{75}$$

For the simple propagation scheme, we can identify $\hat{A} = -(i/\hbar)\Delta t\hat{H}(t)$ and in the atomic

orbital basis

$$\boldsymbol{S}\boldsymbol{\rho}(t+\Delta t)\boldsymbol{S} = \sum_{n=0}^{\infty} \frac{1}{n!}\boldsymbol{S}\boldsymbol{C}_n\boldsymbol{S} \tag{76}$$

$$\boldsymbol{\rho}(t+\Delta t) = \sum_{n=0}^{\infty} \frac{1}{n!}\boldsymbol{C}_n \tag{77}$$

with

$$\boldsymbol{S}\boldsymbol{C}_n\boldsymbol{S} = \frac{-i}{\hbar}\Delta t\left(\boldsymbol{H}(t)\boldsymbol{C}_{n-1}\boldsymbol{S} - \boldsymbol{S}\boldsymbol{C}_{n-1}\boldsymbol{H}(t)\right) \tag{78}$$

$$\boldsymbol{C}_n = \frac{-i}{\hbar}\Delta t\left(\boldsymbol{S}^{-1}\boldsymbol{H}(t)\boldsymbol{C}_{n-1} - \boldsymbol{C}_{n-1}\boldsymbol{H}(t)\boldsymbol{S}^{-1}\right) \tag{79}$$

and with

$$\boldsymbol{C}_0 = \boldsymbol{\rho}(t) \tag{80}$$

More specifically, one would typically define $\boldsymbol{A} = \frac{-i}{\hbar}\Delta t\boldsymbol{S}^{-1}\boldsymbol{H}(t)$ and then write

$$\boldsymbol{C}_n = \boldsymbol{A}\boldsymbol{C}_{n-1} + \boldsymbol{C}_{n-1}\boldsymbol{A}^{\dagger} \tag{81}$$

since both $\boldsymbol{H}(t)$ and $\boldsymbol{S}^{-1}$ are Hermitian.

In practice, we need to introduce a convergence parameter ϵ which truncates the series at smallest n for which $||\boldsymbol{C}_n|| < \epsilon$, assuming uniform convergence.

6 Conservation of Idempotency

As long as the effective Hamiltonian is hermitian, the density operator is idempotent at all times if it is idempotent initially. For generally non-hermitian Hamiltonian, the equation of motion for the density operator would read

$$\frac{\partial \hat{\rho}}{\partial t} = \frac{-i}{\hbar} \left(\hat{H}(t)\hat{\rho}(t) - \hat{\rho}(t)\hat{H}^{\dagger}(t) \right) \tag{82}$$

From this, we can construct equation of motion for $\hat{\rho}^2(t)$

$$\hat{\rho}(t)\frac{\partial \rho}{\partial t} + \frac{\partial \rho}{\partial t}\hat{\rho}(t) = -\frac{i}{\hbar} \left(\hat{\rho}(t)\hat{H}(t)\hat{\rho}(t) - \hat{\rho}^2(t)\hat{H}^{\dagger}(t) + \hat{H}(t)\hat{\rho}^2(t) - \hat{\rho}(t)\hat{H}^{\dagger}(t)\hat{\rho}(t) \right) \tag{83}$$

$$\frac{\partial (\hat{\rho}^2)}{\partial t} = -\frac{i}{\hbar} \left(\hat{H}(t)\hat{\rho}^2(t) - \hat{\rho}^2(t)\hat{H}^{\dagger}(t) \right) - \frac{i}{\hbar}\hat{\rho}(t) \left(\hat{H}(t) - \hat{H}^{\dagger}(t) \right) \hat{\rho}(t) \tag{84}$$

i.e. if at time t, $\hat{\rho}^2(t) = \hat{\rho}(t)$, then such condition is true at all times, as the equation of motion is the same for $\hat{\rho}(t)$ and $\hat{\rho}^2(t)$ at every instant, as long as the Hamiltonian is hermitian.

In the overlapping basis, the form of idempotency condition is given by overlapping resolution of identity (51)

$$\begin{aligned} \langle \phi_\mu | \hat{\rho} | \phi_\nu \rangle = (\boldsymbol{S\rho S})_{\mu\nu} = \langle \phi_\mu | \hat{\rho}^2 | \phi_\nu \rangle = \\ = \sum_{\mu',\nu'} \langle \phi_\mu | \hat{\rho} | \phi_{\mu'} \rangle S_{\mu'\nu'} \langle \phi_{\nu'} | | \phi_\nu \rangle = (\boldsymbol{S\rho S S^{-1} S\rho S})_{\mu\nu} \end{aligned} \tag{85}$$

$$\boldsymbol{\rho} = \boldsymbol{\rho S \rho} \tag{86}$$

In the code, we track the idempotency deviation by outputting

$$\mathrm{Tr}\left(\hat{\rho}^2\right) - \mathrm{Tr}\left(\hat{\rho}\right) = \sum_{\mu} (\boldsymbol{\rho S \rho S})_{\mu\mu} - (\boldsymbol{\rho S})_{\mu\mu} = \sum_{\mu} (\boldsymbol{\rho S}(\boldsymbol{\rho S} - \boldsymbol{I}))_{\mu\mu} \tag{87}$$

7 Hartree and COHSEX Terms in the Gaussian overlapping basis

7.1 Hartree Term

In the position basis, Hartree term is given as

$$\langle \boldsymbol{r} | \hat{V}^{\mathrm{Hartree}}(t) | \boldsymbol{r}' \rangle = V^{\mathrm{Hartree}}(\boldsymbol{r}, \boldsymbol{r}', t) = \delta(\boldsymbol{r} - \boldsymbol{r}') \int d^3 r'' \frac{e^2 \rho(\boldsymbol{r}'', t)}{4\pi\epsilon_0 |\boldsymbol{r} - \boldsymbol{r}''|} \tag{88}$$

where $\rho(\boldsymbol{r}', t) = \langle \boldsymbol{r}' | \hat{\rho}(t) | \boldsymbol{r}' \rangle$. Substituting for covariant representation of the Hartree term in the atomic orbital basis and contravariant representation of the density matrix in the atomic orbital basis, we obtain

$$\sum_{\mu,\nu,\mu',\nu'} \phi_\mu(\boldsymbol{r}) S^{-1}_{\mu\nu} V^{\mathrm{Hartree}}_{\nu\mu'}(t) S^{-1}_{\mu'\nu'} \phi^*_{\nu'}(\boldsymbol{r}') = \langle \boldsymbol{r} | \, \boldsymbol{r}' \rangle \int d^3 r'' \frac{e^2}{4\pi\epsilon_0 |\boldsymbol{r} - \boldsymbol{r}''|} \sum_{\mu,\nu} \phi_\mu(\boldsymbol{r}'') \phi^*_\nu(\boldsymbol{r}'') \rho_{\mu\nu}(t) \tag{89}$$

Multiplying by $\langle \phi_\lambda | \, \boldsymbol{r} \rangle \langle \boldsymbol{r}' | \, \phi_\sigma \rangle$ and integrating in $\boldsymbol{r}$ and $\boldsymbol{r}'$, we obtain

$$V^{\mathrm{Hartree}}_{\lambda\sigma}(t) = \int d^3 r d^3 r' d^3 r'' \langle \phi_\lambda | \, \boldsymbol{r} \rangle \langle \boldsymbol{r} | \, \boldsymbol{r}' \rangle \langle \boldsymbol{r}' | \, \phi_\sigma \rangle \frac{e^2}{4\pi\epsilon_0 |\boldsymbol{r} - \boldsymbol{r}''|} \sum_{\mu\nu} \phi_\mu(\boldsymbol{r}'') \phi^*_\nu(\boldsymbol{r}'') \tag{90}$$

Resolving the completeness relation in $\boldsymbol{r}'$ leads to[1]

$$V^{\mathrm{Hartree}}_{\lambda\sigma}(t) = \sum_{\mu,\nu} \int d^3 r d^3 r'' \phi^*_\lambda(\boldsymbol{r}) \phi_\sigma(\boldsymbol{r}) \frac{e^2}{4\pi\epsilon_0 |\boldsymbol{r} - \boldsymbol{r}''|} \phi^*_\nu(\boldsymbol{r}'') \phi_\mu(\boldsymbol{r}'') \rho_{\mu\nu}(t) = \sum_{\mu,\nu} (\lambda\sigma|\nu\mu) \rho_{\mu\nu}(t) \tag{91}$$

For real orbitals then also $V_{\lambda\sigma}^{\mathrm{Hartree}} = \sum_{\mu\nu} (\lambda\sigma|\mu\nu)\rho_{\mu\nu}$.

7.2 COHSEX Term

The Coulomb hole term has the following position base representation[1]

$$\langle \boldsymbol{r}| \hat{\Sigma}^{\mathrm{COH}}(t) |\boldsymbol{r}'\rangle = -\frac{1}{2} W(\boldsymbol{r}, \boldsymbol{r}', \omega = 0)\delta(\boldsymbol{r} - \boldsymbol{r}') \tag{92}$$

The screened exchange is represented as[1]

$$\langle \boldsymbol{r}| \hat{\Sigma}^{\mathrm{SEX}}(t) |\boldsymbol{r}'\rangle = i\hbar W(\boldsymbol{r}, \boldsymbol{r}', \omega = 0) G^{<}(\boldsymbol{r}, \boldsymbol{r}', t) \tag{93}$$

with

$$\hat{\Sigma}^{\mathrm{COHSEX}}(t) = \hat{\Sigma}^{\mathrm{COH}}(t) + \hat{\Sigma}^{\mathrm{SEX}}(t) \tag{94}$$

where $G^{<}(\boldsymbol{r}, \boldsymbol{r}', t)$ is the time-diagonal lesser Green's function.[1]

Under the approximation that the screened exchange W does not change as time passes, the Coulomb hole part of the self-energy is constant, and hence does not contribute to the dynamics.[1]

The screened exchange part can be expressed in terms of the density matrix, since

$$G^{<}(\boldsymbol{r}, \boldsymbol{r}', t) = \frac{i}{\hbar} \rho(\boldsymbol{r}, \boldsymbol{r}', t) \tag{95}$$

leading to

$$\sum_{\mu,\nu,\mu',\nu'} \phi_{\mu}(\boldsymbol{r}) S^{-1}_{\mu\nu} \Sigma^{\mathrm{SEX}}_{\nu\mu'} S^{-1}_{\mu'\nu'} \phi^{*}_{\nu'}(\boldsymbol{r}') = -W(\boldsymbol{r}, \boldsymbol{r}', \omega = 0) \langle \boldsymbol{r}| \hat{\rho}(t) |\boldsymbol{r}'\rangle \tag{96}$$

Similarly as before, multiplying by $\langle \phi_\lambda | \, \boldsymbol{r} \rangle \langle \boldsymbol{r'} | \, \phi_\sigma \rangle$ and integrating in $\boldsymbol{r}$ and $\boldsymbol{r'}$,[1]

$$\Sigma^{\text{SEX}}_{\lambda\sigma}(t) = -\int d^3r \int d^3r' \sum_{\mu,\nu} \phi^*_\lambda(\boldsymbol{r})\phi_\mu(\boldsymbol{r})W(\boldsymbol{r},\boldsymbol{r'},\omega=0)\phi_\sigma(\boldsymbol{r'})\phi^*_\nu(\boldsymbol{r'})\rho_{\mu\nu}(t) \tag{97}$$

$$\Sigma^{\text{SEX}}_{\lambda\sigma}(t) = -\sum_{\mu,\nu} W_{\lambda\mu,\nu\sigma}\rho_{\mu\nu}(t) \tag{98}$$

where we used the $W_{\lambda\mu,\nu\sigma}$ notation for the screened four-centre integral.

8 RI Approximation

Resolution of identity approximation can be used to reduce the scaling of electron repulsion integrals with number of basis functions. The basic idea is that the basis pair products are substituted by summations over auxiliary basis functions which minimize difference from true ERIs, specifically[12]

$$\phi_\mu(\boldsymbol{r})\phi_\nu(\boldsymbol{r}) = \sum_P B^P_{\mu\nu}\phi_P(\boldsymbol{r}) \tag{99}$$

where $B^P_{\mu\nu} = \sum_Q m^{-1}_{PQ}(Q][\mu\nu)$, where $(Q][\mu\nu)$ is a 3-centre integral with $m(\boldsymbol{r},\boldsymbol{r'})$ as the metric.

This simplifies the calculation of Hartree term to

$$V^{\text{Hartree}}_{\lambda\sigma} = \sum_{P,Q}(\lambda\sigma][P)(\boldsymbol{m}^{-1}\boldsymbol{V}^{\text{H,A}}\boldsymbol{m}^{-1})_{PQ}\sum_{\mu,\nu}(Q][\nu\mu)\rho_{\mu\nu}(t) \tag{100}$$

where

$$V^{\text{H,A}}_{PQ} = \int d^3r d^3r' \phi^*_P(\boldsymbol{r})\frac{1}{|\boldsymbol{r}-\boldsymbol{r'}|}\phi_Q(\boldsymbol{r'}) \tag{101}$$

Similar approximation can be made for the screened exchange self-energy, with

$$\Sigma^{\mathrm{SEX}}_{\lambda\sigma}(t) = -\frac{1}{\hbar}\sum_{P,Q,\mu,\nu}(\lambda\mu][P)(\boldsymbol{m}^{-1}\boldsymbol{W}^{\mathrm{A}}\boldsymbol{m}^{-1})_{PQ}(Q][\nu\sigma)\rho_{\mu\nu}(t) \tag{102}$$

9 RI Auxiliary Basis Set Convergence

We varied the RI auxiliary basis set for a fixed orbital basis set (aug-cc-pVDZ), specifically, we used aug-cc-pVDZ-RIFIT (DZ), aug-cc-pVTZ-RIFIT (TZ), aug-cc-pVQZ-RIFIT (QZ) and aug-cc-pV5Z-RIFIT (5Z). The recorded LUMO-HOMO (lowest unoccupied/highest occupied molecular orbital) gap, as recovered from the GW, are shown below in Fig. S1, with respect to the gap observed for 5Z calculation.

10 RI Cutoff Radius Convergence

We varied the cutoff-radius of the RI-metric used for the 3-centre integrals in RI approximation to study the effect on the LUMO-HOMO gap, shown Fig. S2.

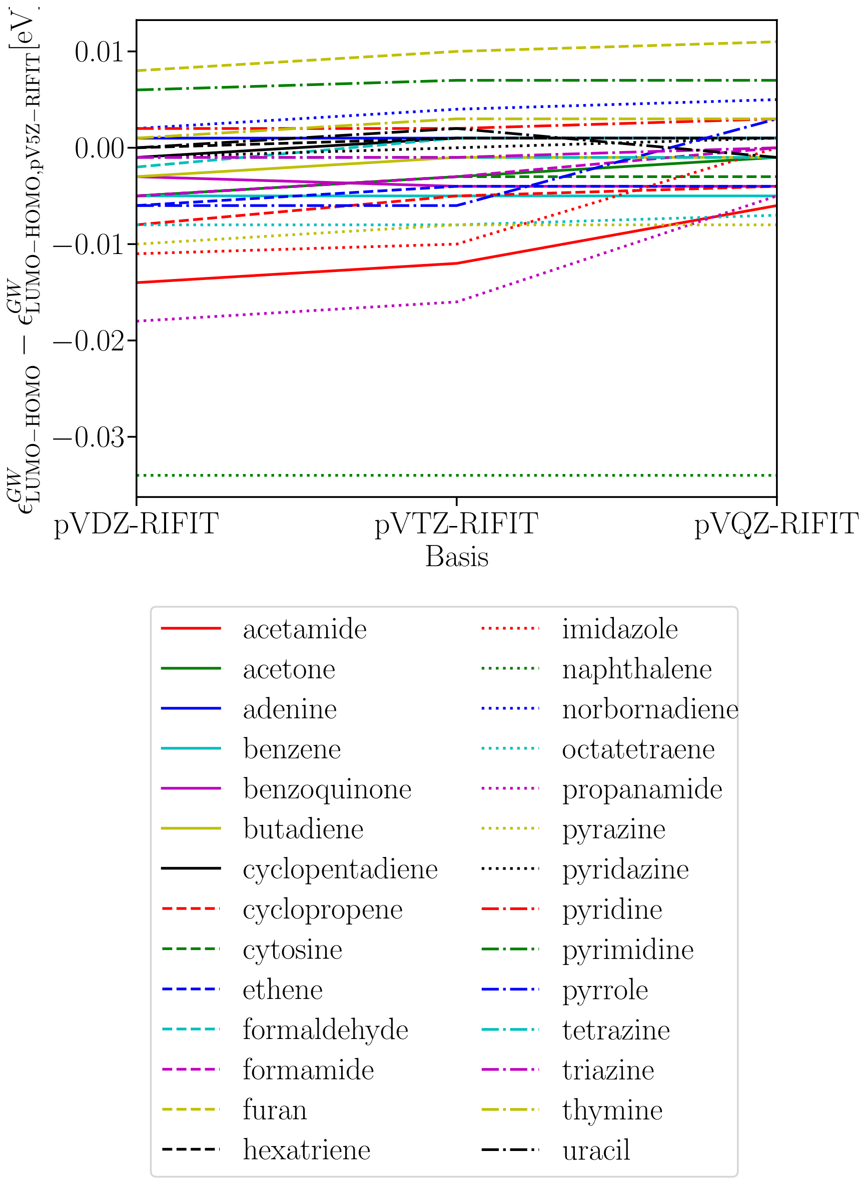

Figure S1: LUMO-HOMO gap of molecules in the Thiel's set with differing auxiliary basis set. The variation with respect to 5Z is around 10 meV, going up to about 30 meV for largest deviation.

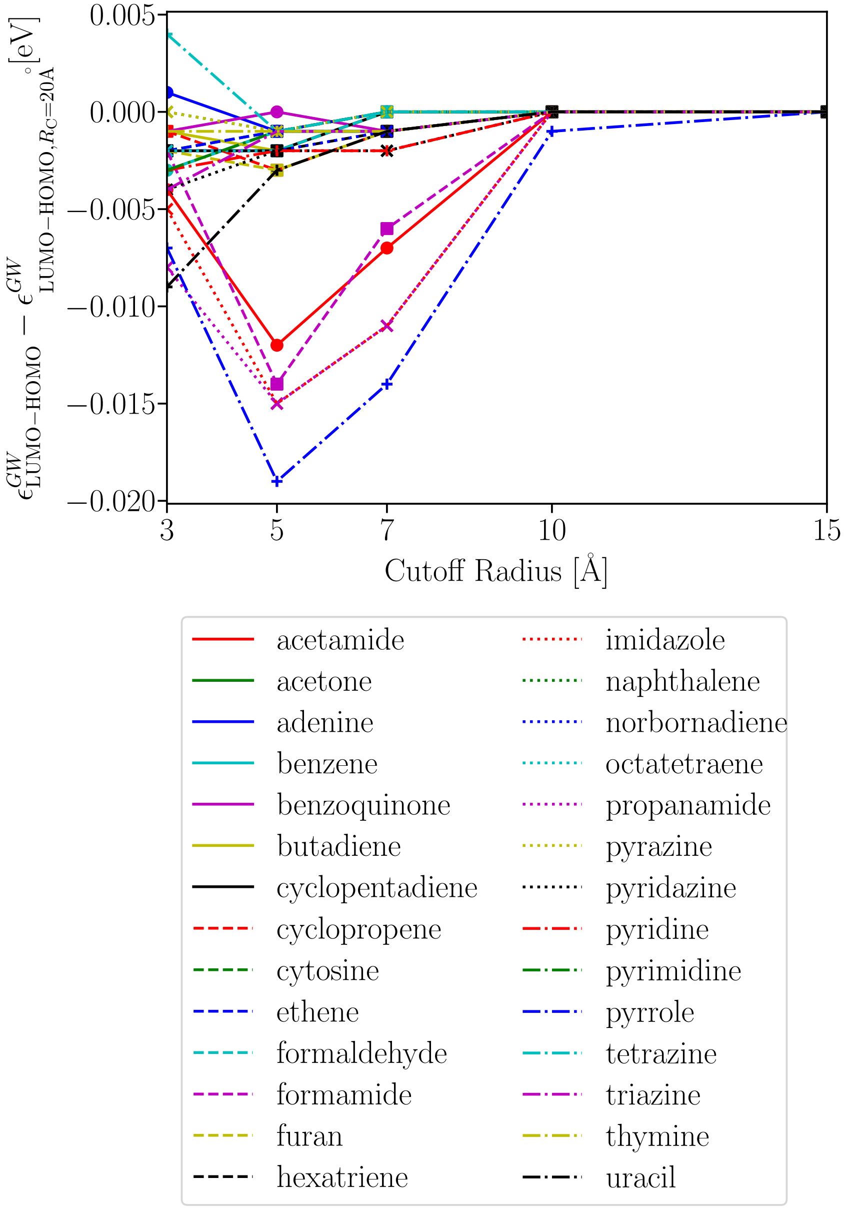

Figure S2: LUMO-HOMO gap of molecules in the Thiel's set with different RI cutoff radius. The RI basis used here was the aug-cc-pvtz-rifit. The values are compared to a gap with 20 Å cutoff radius. The values start to converge from 7 Å used in the main text.

11 Timestep Convergence in HHG of Cysteine

We checked a smaller timestep of 0.5 as, which showed no divergence from the 1 as timestep used in the main body of the paper. The time series of the oscillating dipole moment as well as the Fourier transform using Gaussian window function are shown in Fig. S3.

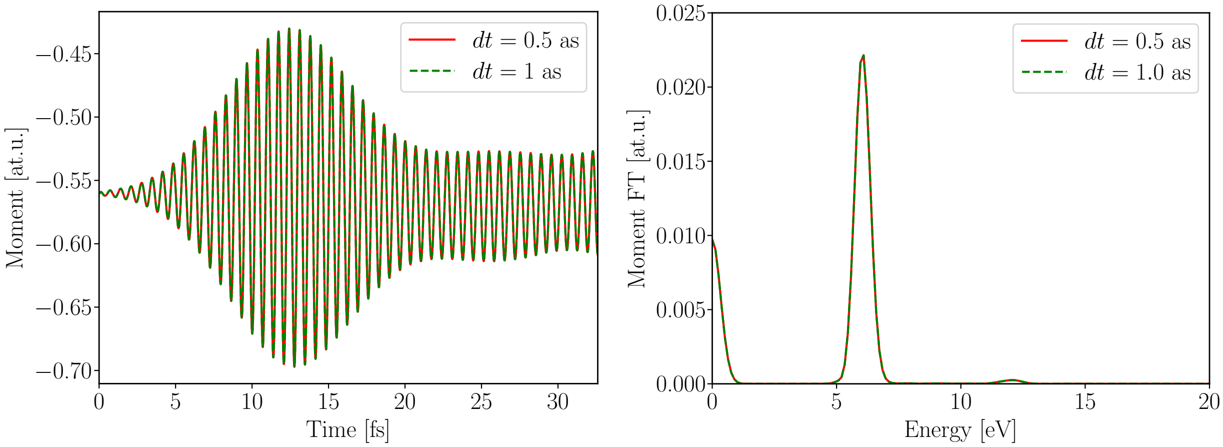

Figure S3: On the left, the oscillating dipole moment series in cysteine for different size of the timestep in the simulation. No visible departure within the investigated times is observed. On the right, Fourier transform of the series using Gaussian window function centred at 22.6 fs with 2.0 fs spread. Again, no visible difference is observed between the two spectra.

Since no visible difference is present in either the time series or in the spectra, we conclude that the simulation is converged in the timestep size.

12 Comparison of Spectra with and without Padé Interpolation

The Padé interpolation is used to increase the density of sampling of the Fourier transform of the dipole moment time series. Comparison between the simple Fourier transform and the Padé refined spectra fitted to the transform is presented in Fig. S4.

Padé interpolation also increases consistency of spectra obtained from different propagation times, as we illustrate in Fig. S5.

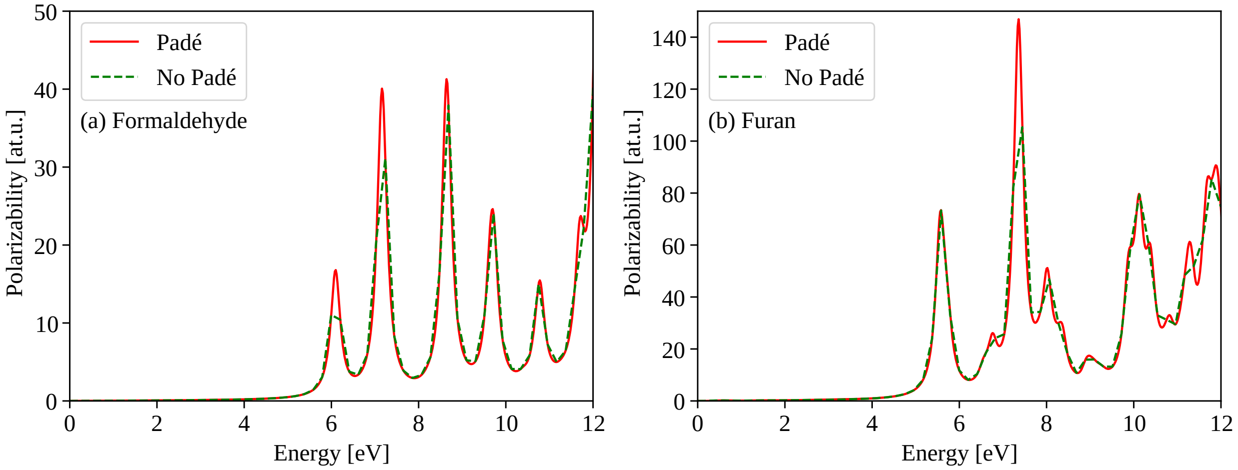

Figure S4: The formaldehyde (a) and furan (b) spectra with (solid line) and without (dashed line) Padé interpolation. The interpolation allows for arbitrary density of sampled points in the energy space, effectively allowing super-resolution of the spectrum, which is otherwise limited by the total propagation time of 20 fs to about 0.2 eV.

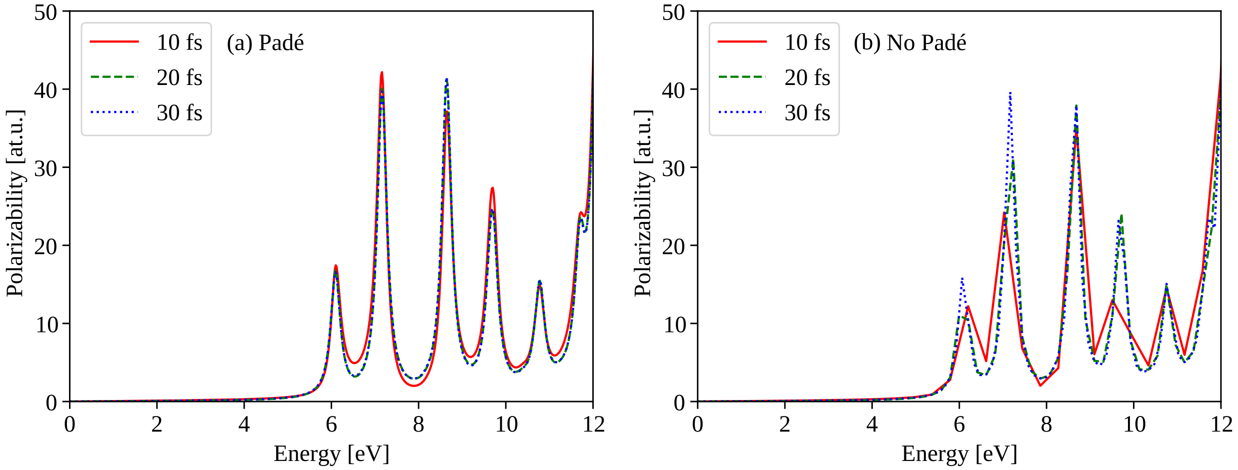

Figure S5: Comparison of spectra obtained for different total propagation times with (a) Padé interpolation and without (b) Padé interpolation. Besides projecting the spectra on equivalent energy grids, the consistency of the peaks is increased when using Padé interpolation.

13 Illustration of Low-Scaling vs Standard-Scaling Eigenvalue difference

In Table S1, we show the difference between the eigenvalues from low-scaling and standard-scaling G_0W_0 implementations in CP2K for the first few eigenvalues of formaldehyde. Note that the difference increases for orbitals far away in energy from the HOMO/LUMO orbitals. We attribute the rather large deviation of RT-BSE (starting from low-scaling GW eigenvalues) and LR-BSE (starting from standard-scaling GW eigenvalues) for formaldehyde (Fig. 1a in the main text) for peaks with peak energy > 8 eV to the error 0.2 eV in HOMO–1 (7) in low-scaling GW.

Table S1: Index of the molecular orbital and its energy in the two G_0W_0 implementations in CP2K, with low-scaling values used for the presented implementation of RT-BSE and standard scaling values used for the LR-BSE implementation.

Eigenvalue index	Low-scaling $\epsilon^{G_0W_0}$ [eV]	Standard-scaling $\epsilon^{G_0W_0}$ [eV]	Difference [eV]
1	-588.541	-565.971	7.43
2	-306.012	-300.845	-5.17
3	-38.020	-32.409	-5.61
4	-20.761	-20.630	-0.131
5	-16.532	-16.443	-0.089
6	-15.307	-15.287	-0.020
7	-13.877	-13.663	-0.214
8 (HOMO)	-10.235	-10.245	-0.010
9 (LUMO)	1.503	1.503	0.000
10	0.936	0.940	-0.004
11	1.442	1.447	-0.005
12	2.315	2.316	-0.001
13	2.911	2.911	0.000
14	4.108	4.113	-0.005
15	4.023	4.024	-0.001
16	5.733	5.731	0.002
17	6.037	6.034	0.003
18	6.891	6.892	0.001
19	6.678	6.668	0.010
20	8.060	8.049	0.011

14 Computational Scaling

We investigated the scaling of the execution time of the most expensive routine `get_sigma` (determination of the screened exchange self energy) of the code as a function of the basis set size. The routine is called many times during the execution of the code – we report the average timing per one call. Typically, the routine is called about 2-5 times per timestep and the propagation in the validation runs used 20 000 time steps. We observed a $T \propto N^{3.1 \pm 0.2}$ dependence, as shown in Fig. S6.

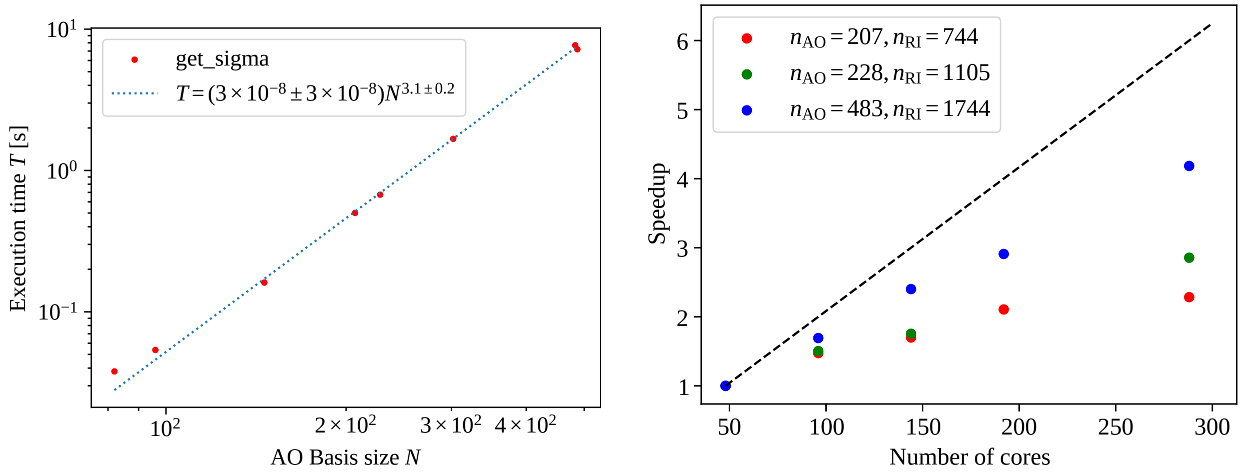

Figure S6: Left: Scaling of the execution time of the most expensive routine in the calculation. We observe approximately cubic scaling with the size of the basis. All data use 16 MPI ranks, each with 3 OMP threads. Right: Strong scaling investigation – for a fixed basis set size, we increase the number of cores and note the speedup. We see that the speedup plateaus later for larger systems, hinting at possible presence of weak scaling phenomenon. The dashed line shows perfect parallelisation speedup.

The N^3 scaling is slower than anticipated from (102), where the most expensive steps are expected to have N^4 scaling (for example contraction of $\sum_\mu (Q][\nu\mu)\rho_{\mu\nu}$). The reduced scaling shows that the parallel efficiency may not yet be fully realized for the number of cores used in the test.

The dependence of the maximum attainable speedup on the system size shows the possibility of weak scaling phenomenon, which we decided to further validate. We ran a series of calculations where the computational effort (cube of the basis set size, N^3) increased

approximately along with the number of resources used for the calculation (number of computational nodes, each with 48 processors). This produced series of average times required per call of `get_sigma`, $T_N(N)$. We also calculated the average time required per call of the `get_sigma` routine when running on a single node for all of these cases, $T_N(1)$. The chosen parameters are shown in Table S2, while the speedup $T_N(1)/T_N(N)$ is shown in Fig. S7.

Table S2: The number of nodes and the approximately proportional problem size used for the demonstration of Gustafson's law in Fig. S7.

# Nodes	N	N^3
1	146	3.11×10^6
2	183	6.13×10^6
3	210	9.26×10^6
4	228	11.85×10^6
6	261	17.78×10^6
8	302	27.54×10^6

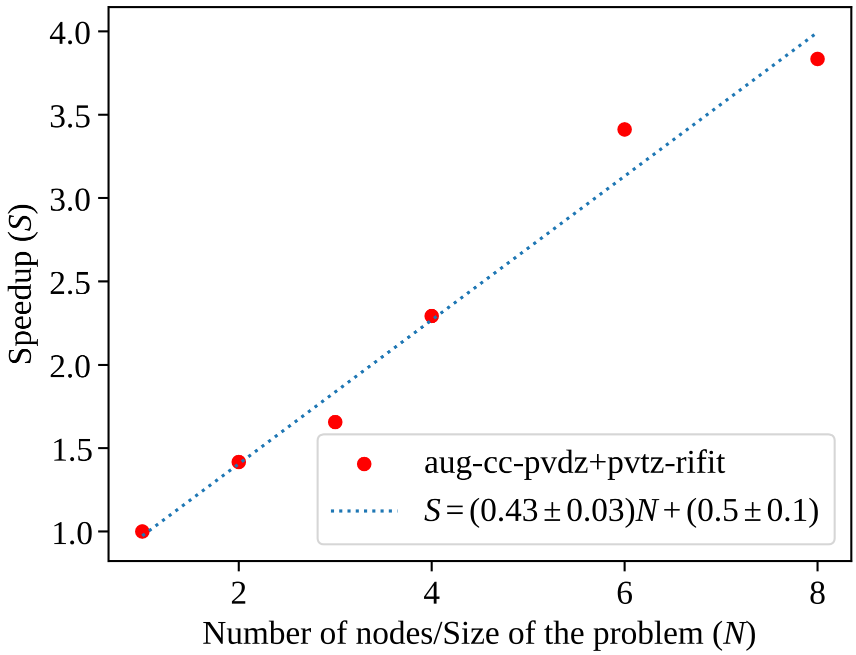

Figure S7: The speedup obtained for system size increasing approximately together with the number of cores used for the calculation. The approximately linear dependence is consistent with Gustafson's law.[13]

We also evaluated the computational scaling of the storage of the most expensive object – the three centre integrals $(Q|\nu\mu)$. The storage grows as N^3, as shown in Fig. S8. Note

that for small system sizes, the actual memory required for the RT-BSE run will be larger, but for larger systems the contribution from the 3 centre integrals will become dominant.

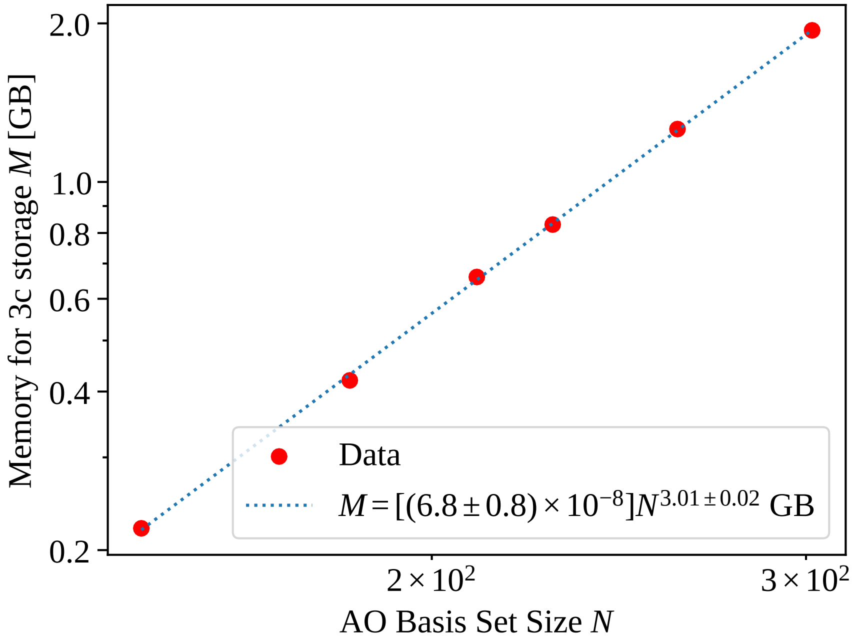

Figure S8: Memory required for the storage of 3 centre integrals, growing as N^3 with the system size, as expected.

References

(1) Attaccalite, C.; Grüning, M.; Marini, A. Real-time approach to the optical properties of solids and nanostructures: Time-dependent Bethe-Salpeter equation. *Phys. Rev. B* **2011**, *84*, 245110.

(2) Liu, C.; Kloppenburg, J.; Yao, Y.; Ren, X.; Appel, H.; Kanai, Y.; Blum, V. All-electron ab initio Bethe-Salpeter equation approach to neutral excitations in molecules with numeric atom-centered orbitals. *J. Chem. Phys.* **2020**, *152*, 044105.

(3) Sander, T.; Kresse, G. Macroscopic dielectric function within time-dependent density functional theory—Real time evolution versus the Casida approach. *J. Chem. Phys.* **2017**, *146*, 064110.

(4) Mattiat, J.; Luber, S. Comparison of Length, Velocity, and Symmetric Gauges for the Calculation of Absorption and Electric Circular Dichroism Spectra with Real-Time Time-Dependent Density Functional Theory. *J. Chem. Theory Comput.* **2022**, *18*, 5513–5526.

(5) Castro, A.; Marques, M. A. L.; Rubio, A. Propagators for the time-dependent Kohn–Sham equations. *J. Chem. Phys.* **2004**, *121*, 3425–3433.

(6) Müller, C.; Sharma, M.; Sierka, M. Real-time time-dependent density functional theory using density fitting and the continuous fast multipole method. *J. Comput. Chem.* **2020**, *41*, 2573–2582.

(7) Cistaro, G.; Malakhov, M.; Esteve-Paredes, J. J.; Uría-Álvarez, A. J.; Silva, R. E.; Martín, F.; Palacios, J. J.; Picón, A. Theoretical approach for electron dynamics and ultrafast spectroscopy (edus). *J. Chem. Theory Comput.* **2022**, *19*, 333–348.

(8) Wu, M.; Chen, S.; Camp, S.; Schafer, K. J.; Gaarde, M. B. Theory of strong-field attosecond transient absorption. *J. Phys. B:At., Mol. Opt. Phys.* **2016**, *49*, 062003.

(9) Yabana, K.; Nakatsukasa, T.; Iwata, J.-I.; Bertsch, G. F. Real-time, real-space implementation of the linear response time-dependent density-functional theory. *Phys. Status Solidi B* **2006**, *243*, 1121–1138.

(10) Martin, R. M. *Electronic Structure: Basic Theory and Practical Methods*, 2nd ed.; Cambridge University Press: Cambridge, 2020.

(11) O'Rourke, C.; Bowler, D. R. Linear scaling density matrix real time TDDFT: Propagator unitarity and matrix truncation. *J. Chem. Phys.* **2015**, *143*, 102801.

(12) Graml, M.; Zollner, K.; Hernangómez-Pérez, D.; Faria Junior, P. E.; Wilhelm, J. Low-Scaling GW Algorithm Applied to Twisted Transition-Metal Dichalcogenide Heterobilayers. *J. Chem. Theory Comput.* **2024**, *20*, 2202–2208.

(13) Gustafson, J. L. Reevaluating Amdahl's law. *Commun. ACM* **1988**, *31*, 532–533.
